# Mining and Mapping 25 Years of Medication Use in Child and Adolescent Mental Health Services: Contact-Level Descriptive Analysis of Electronic Health Records

**DOI:** 10.2196/86066

**Published:** 2026-06-16

**Authors:** Dipendra Pant, Carolyn Clausen, Bennett L Leventhal, Thomas Brox Røst, Roman Koposov, Odd Sverre Westbye, Jostein Arntzen, Norbert Skokauskas, Øystein Nytrø

**Affiliations:** 1 Department of Computer Science Norwegian University of Science and Technology Trondheim, Trøndelag Norway; 2 Department of Child and Adolescent Psychiatry Clinic of Mental Health Care St Olav's University Hospital Trondheim, Trøndelag Norway; 3 Department of Mental Health Regional Centre for Child and Youth Mental Health and Child Welfare (RKBU Central Norway) Norwegian University of Science and Technology Trondheim, Trøndelag Norway; 4 Psychiatry and Behavioral Neuroscience The University of Chicago Chicago, IL United States; 5 Regional Centre for Child and Youth Mental Health and Child Welfare (RKBU North) UiT The Arctic University of Norway Tromsø, Troms Norway; 6 Department of Computer Science UiT The Arctic University of Norway Tromsø, Troms Norway

**Keywords:** children, adolescents, mental health, data mining, electronic health records, medication, diagnosis, knowledge representation, clinical decision support

## Abstract

**Background:**

Norwegian Child and Adolescent Mental Health Services (CAMHS) use the World Health Organization’s (WHO) multiaxial diagnostic system based on the *International Classification of Diseases, Tenth Revision* (ICD-10); however, analysis of prescribing patterns among axes I-III is underexplored in electronic health records (EHRs) with intertwined patient, episode of care, and contact information.

**Objective:**

This study aimed to develop and demonstrate an analytic pipeline for mining and mapping information from EHRs to facilitate understanding of clinical processes and support informed decision-making. This study used the Norwegian CAMHS EHR data to identify common diagnoses, comorbidities, and medication use across axes I-III per individual contact.

**Methods:**

We extracted records of patients ≤19 years old with a primary mental health diagnosis on axes I-III and one or more medications per individual contact. Diagnoses were categorized according to ICD-10 and medications according to the Anatomical Therapeutic Chemical (ATC) classification system. Descriptive analyses quantified contact counts, diagnosis frequency, comorbidity rates, and medication frequency within each diagnostic category. Next, we mapped the medications used across all the contacts and noncomorbid contacts separately along each axis.

**Results:**

Of 7214 prescribing contacts (axis I: n=7179, 99.51%; axis II: n=821, 11.38%; axis III: n=65, 0.90%), comorbidity was present in 12.06% (n=866) contacts in axis I, 96.10% (n=789) contacts in axis II, and 96.92% (n=63) contacts in axis III. Leading diagnoses were behavioral-emotional disorders (ICD-10 codes F90-F98) in axis I, school skills and learning difficulties (ICD-10 code F81) in axis II, and mild mental retardation (ICD-10 code F70) in axis III. Most observed comorbidities were F90-F98 with speech and language development disorder (ICD-10 code F80), ICD-10 code F81, and mixed specific skills development disorder (ICD-10 code F83). Psychostimulants predominated across all diagnosis axes, with methylphenidate being the most common. For other ATC categories, the most commonly prescribed medications were antidepressants (sertraline and fluoxetine), antipsychotics (risperidone and aripiprazole), hypnotics and sedatives (melatonin), antiepileptics (lamotrigine), anxiolytics (diazepam), and nonpsychotropics (laxatives, vitamins, and supplements). Medication profiles varied minimally by axis or comorbidity status.

**Conclusions:**

We demonstrated a mining and mapping analytic pipeline for EHRs to analyze diagnoses, comorbidities, and prescribing practices at the individual contact level. In the Norwegian CAMHS, axis I diagnoses are common, often behavioral-emotional disorders. Among the medications, psychostimulants and antidepressants are common. Beyond characterizing diagnoses and medication prescribing patterns, the study presents an approach for mining and mapping EHR data to analyze and provide service-level metrics, as well as clinical practice insights.

## Introduction

Norwegian Child and Adolescent Mental Health Services (CAMHS), like many CAMHS worldwide, use the *International Classification of Diseases, Tenth Revision* (ICD-10) for diagnostic purposes [1]. Norwegian CAMHS use a national adaptation of the World Health Organization’s (WHO) ICD-10 multiaxial framework, where different aspects are grouped to provide a useful and unambiguous view of the patient’s disorder in CAMHS [2]. The axis for the psychosocial situation is coded with a locally relevant classification, and the function axis uses the Children’s Global Assessment Scale (CGAS). Medication and prescriptions are coded using the Anatomical Therapeutic Chemical (ATC) classification system developed by the WHO [3]. In the WHO multiaxial framework, axis I covers clinical psychiatric syndromes, axis II covers specific developmental disorders, and axis III covers intellectual disability (mental retardation) [1,2,4]. This multiaxial framework aims to provide a clinically useful picture of mental health, distinguishing, for example, an expressive language disorder on axis II from a comorbid attention deficit/hyperactivity disorder (ADHD) on axis I or noting an intellectual disability (mental retardation) on axis III [1,2]. Such differentiation is clinically important, as it guides individualized treatment planning across psychiatric, developmental, and cognitive domains. However, despite its structural design, prescribing patterns and comorbidity profiles across WHO multiaxial axes I-III remain underexplored, and mining and mapping could provide insights into diagnostic patterns and clinical decision-making.

The ATC classification system organizes medications according to their anatomical site of action, therapeutic use, and pharmacological properties [3,5]. This allows mapping of psychotropic and nonpsychotropic medication categories and enables systematic analysis across diagnostic categories. Medications are pharmaceutical products, or drugs, intended for administration to patients, and the use of psychotropic medications in child and adolescent mental health care has expanded over the past two decades worldwide [6]. Previous international studies have reported an increase in the number of medication prescriptions, including stimulants for ADHD and antidepressants for adolescent mood/anxiety disorders [6]. For instance, from 2000 to 2019, stimulant use roughly doubled in England [7]. In Norway, and other Nordic countries, the diagnosis of ADHD and associated treatment involving stimulant medications has risen markedly in the 2000s [8,9]. It is estimated that around 7% of children aged 4-14 years in Norway have a diagnosable mental disorder, while about 1 in 5 adolescent girls and 1 in 10 boys report experiencing multiple psychological problems [10], and medications are now an important component of treatment for moderate-to-severe psychiatric conditions. Despite this general trend, the use of medication across different diagnostic categories in routine practice is not fully explored. Worldwide, most pharmacoepidemiological research in child and adolescent psychiatry focuses on single disorders in isolation or broad nationwide prescribing rates, rather than examining differences by diagnostic axis [7,11,12].

**Figure 1:**
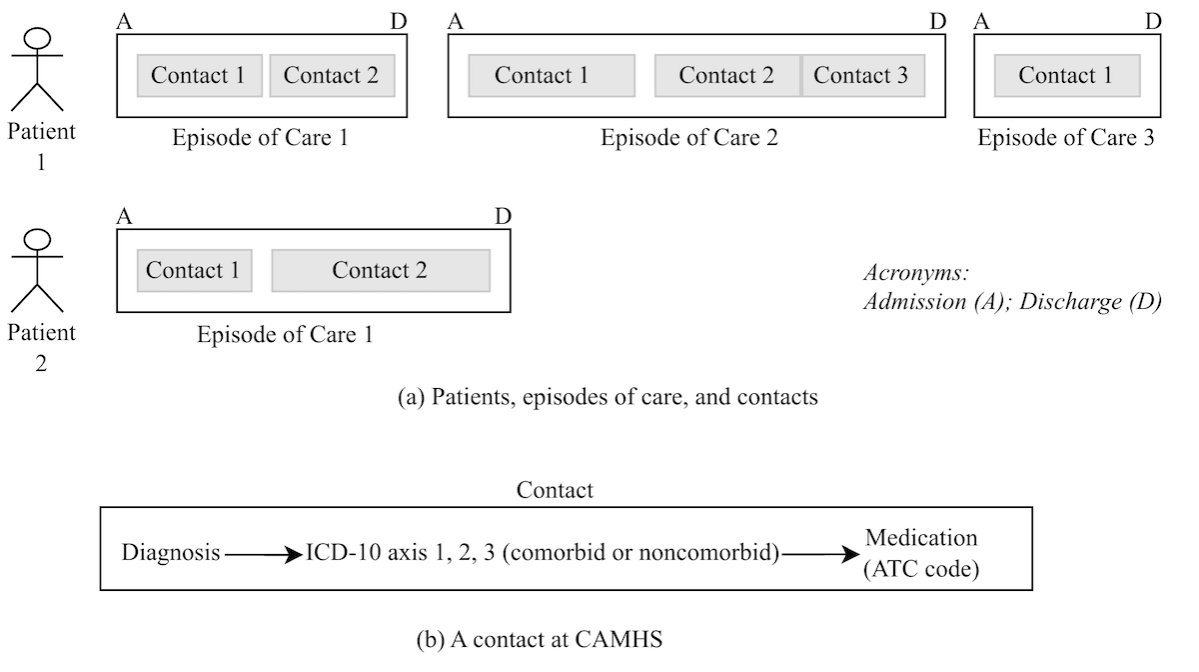
Patients, episodes of care, and contacts at CAMHS. ATC: Anatomical Therapeutic Chemical; CAMHS: Child and Adolescent Mental Health Services; ICD-10: *International Classification of Diseases, Tenth Revision*.

In Norwegian CAMHS electronic health records (EHRs), clinicians have historically recorded multiaxial diagnoses for each patient in line with the multiaxial classification system; however, research to date neither relies on secondary use of EHRs [13] nor uses such data to examine the use of psychotropic medications and comorbidities across the axes. For this study, a Norwegian CAMHS EHR [4,14] containing information at several interconnected levels about patients, their episodes of care, and individual contacts (encounters), capturing different aspects of clinical activity, was used [4]. In this study, an “episode of care” was a defined period during which a patient receives continuous mental health services for a specific issue by one responsible service unit (ie, hospital or clinic), starting with an accepted referral, followed by a decision to provide care or an acute admission, and ending with a formal discharge; no additional time window rule was used to merge or split episodes. Each episode of care included one or more contacts (encounters) with the patient (see Figure 1a). A contact was any single, recorded interaction between the patient and the care team within an episode of care (see Figure 1b), and each contact contained information about diagnosis, as well as prescribed medications. Diagnoses or prescriptions documented on separate contacts were retained as separate contact-level observations.

Using the CAMHS EHR data described in the *Data Source and Study Cohort* section later, the analytic pipeline presented clinical insights and decision-making related to multiaxial diagnostic behavior and prescribing patterns. Specifically, we presented the demographic and clinical profiles of medicated children and adolescents by diagnostic axis, identified the proportions of diagnoses and prescribed medications, mapped medication prescriptions (by ATC category) to their corresponding diagnostic categories, and determined comorbid diagnoses across axes. The data pipeline extracted, preprocessed, mapped, and visualized diagnostic and medication proportions, with individual clinical contacts as the unit of analysis. The study focused on the following research questions (RQs):

- RQ1: What are the proportions of diagnoses and prescribed medications among patient contacts with a primary axis I, II, and III diagnosis?
- RQ2: Which comorbid conditions are observed among patient contacts with a primary axis I, II, and III diagnosis?
- RQ3: Which medications are prescribed among patient contacts for each primary axis I, II, and III diagnosis, both including and excluding comorbidities?

## Methods

### Data Source and Study Cohort

This study used EHR data from a part of the Central Norway Regional CAMHS, which include all specialist care inpatient and outpatient contacts. These specialized, publicly funded CAMHS clinics focus exclusively on the treatment of child and adolescent mental disorders. These regional EHR data do not include nationwide patient coverage. The available EHR contains a total of 22,643 patients, encompassing 30,938 episodes of care and 41,411 contacts with child and adolescent patients, with a “contact” being defined as the smallest record unit, corresponding to a single encounter between a patient and a service provider within an episode of care (as illustrated in Figure 1a). The EHR extract did not include free-text notes, behavioral narratives, and patient-reported data. For all 41,411 contacts, only 22.17% included a medication prescription. The EHR data contained episodes of care beginning in July 1982. For this study, we restricted the cohort to episodes of care that started and ended after 1995 because 1685 patients, with 897 contacts, were from before this date (including medicated and unmedicated), and applying the additional study filters would have further reduced that early cohort. This restriction allowed us to focus on the systematic, analytically stable portion of the available extract; it was not intended to be a representative sample of the population and did not include untreated or unmedicated CAMHS contacts as a comparison group. We acknowledge that these excluded episodes spanned the 1995 boundary or were still ongoing at the time of data extraction.

Accordingly, the study filtered child and adolescent contacts from the EHR that fulfilled all three inclusion criteria: (1) patients aged 19 years or younger at the time of episode start; (2) patient contacts with a recorded ICD-10-coded primary diagnosis on axis I, II, or III; and (3) at least one medication prescription associated with the patient contact. These inclusion criteria were used to preprocess the EHR records, in line with the RQs. The medicated contacts facilitated mapping medication use to diagnostic axes; consequently, the findings visualize prescribing practices and do not represent the broader prevalence of these conditions in the general or unmedicated CAMHS population.

We aggregated the data at the patient level for patients with multiple episodes and then extracted demographic summaries (ie, gender, language, father’s and mother’s ethnicity, and parent relationship type), as these are permanent. This patient demographic information is specific to each patient. At the same time, medication prescriptions and diagnoses were analyzed at the individual contact level to capture the proportions of occurrences. Age was derived from the patient’s date of birth and the start of the episode of care, which was available in the EHR (ie, at the episode level). The diagnosis and prescribed medication were linked to individual contacts; therefore, the overall analysis was based on the contact level, with the contact as the unit of analysis. Data extraction and processing were conducted to derive the dataset variables (see Multimedia Appendix 1) by joining and mapping information about the patient, episode of care, contact, medication, and diagnosis (see the database schema in Multimedia Appendix 2).

We concentrated on mental disorder diagnoses and the corresponding prescribed medications for treatment based on these diagnoses. The analysis was limited to axes I-III, with axes IV-VI excluded. This is because we did not have access to prescriptions or medication information originating outside the CAMHS clinic for disorders that axes IV-VI may cover. Somatic diseases coded on axis IV were often diagnosed and treated by health care professionals and services outside of CAMHS. Consequently, comorbidity in this study referred to co-recorded CAMHS mental health primary diagnoses within axes I-III and did not include somatic or other non-CAMHS conditions. The patients and their contacts were stratified into three groups by their primary diagnosis in each axis, following the WHO multiaxial framework [1,2] used in the Norwegian CAMHS, as follows:

- Axis I: clinical psychiatric syndromes (eg, depressive disorders, anxiety disorders, behavioral and hyperkinetic disorders, psychosis, typically corresponding to ICD-10 codes F03-F48, F50-F69, F84, F90-F99, R40-R46, and Z00-Z71)
- Axis II: specific developmental disorders (eg, developmental language disorder, school skills and learning difficulties, and other developmental disorders, typically corresponding to ICD-10 codes F80-F83 and F88-F89)
- Axis III: mental development disability or intellectual developmental disorders (eg, mild, moderate, or severe intellectual disability, typically corresponding to ICD-10 codes F70-F71 and F79).

We excluded diagnoses that did not represent actual clinical conditions after consultation with the CAMHS clinicians. In particular, diagnosis codes that could not be decoded or indicated administrative entries were excluded (codes 000 or 999 on all axes). We also excluded codes 1000 and 1999, as well as codes X6n, Z724, and F710 (rarely occurring and wrongly labeled; could not be verified as corresponding to ICD-10 axis III intellectual disability and appeared to represent administrative entries or invalid diagnoses) when they appeared as primary diagnoses in axis I; codes 2000 and 2999 in axis II; and codes 3000 and 3999, as well as CGAS codes 1-5, 9, and 99 in axis III (since these do not denote a confirmed disorder but rather CGAS scores). The exclusion applied only to code F710 when it was recorded as the primary diagnosis on axis I; legitimate code F71 entries recorded as the primary diagnosis on axis III were considered valid and retained. The CGAS codes exist in axis VI [1], but in our EHR data, when axis III contained CGAS codes 1-5, 9, and 99, these were also excluded. Codes Z00-Z71 (factors affecting health status and contact with the health service) and R40-R46 (symptoms and signs associated with cognition, perception, emotional state, and behavior) were used in practice when a child was under evaluation or for follow-up contact. A total of 7214 contacts (ie, 3415 patients) satisfied the filtering criteria (ie, age ~19 years or younger; episode-of-care starting and ending after 1995; recorded primary diagnosis on axis I, II, or III, excluding the aforementioned codes; and at least one prescribed medication) and hence were used in this study. The interventions in excluded contacts were outside the scope of this study.

### Measures

#### Diagnoses

Diagnostic codes in the dataset were based on ICD-10 and assigned by CAMHS clinicians during clinical contact with patients, rather than by episode of care. Data were drawn from the clinician-maintained working record in the EHR. For each contact, we mapped the primary key linking the diagnosis. During each contact, one or more coded diagnoses could be recorded across the same or different axes. The structured extract available to us did not support reliable use of uncoded narrative information, so the analysis was limited to coded diagnoses linked to each contact. For the analysis, we focused on the primary diagnosis in each contact. If multiple primary diagnoses were found to be recorded within the same contact, we treated them as a comorbid contact. This operational definition reflects co-recorded CAMHS mental health diagnoses within the structured EHR and likely underestimates broader clinical comorbidity, including uncoded secondary diagnoses and somatic conditions outside axes I-III. If only one primary diagnosis was recorded within a contact, they were treated as a noncomorbid contact. Multiple primary diagnoses recorded within a contact across different axes (eg, an axis I disorder together with an axis II disorder) were classified as comorbid contacts. We grouped each diagnosis code in the CAMHS dataset by category (see Table 1) following the multiaxial framework used in the Norwegian CAMHS [1,2], tabulated the contact counts, identified and ranked the comorbid diagnosis categories, and mapped the corresponding prescribed medications.

**Table 1:** Observed and categorized ICD-10^a^ codes occurring in the Norwegian CAMHS^b^ dataset.

Axis and ICD-10 code/code range		Description
**Axis I**		
	F03-F09	Organic, symptomatic
	F10-F19	Psychoactive substances
	F20-F29	Schizophrenia and paranoia
	F30-F39	Affective/mood disorders
	F40-F48	Neurotic and somatoform disorders
	F50-F59	Behavioral syndromes
	F60-F69	Personality and behavioral disorders
	F84	Pervasive developmental disorders
	F90-F98	Childhood behavioral and emotional disorders
	F99	Unspecified mental disorder
	R40-R46	Cognition and emotional signs
	Z00-Z71	Health services contacts
**Axis II**		
	F80	Speech and language development disorders
	F81	School skills and learning difficulties
	F82	Motor skills disorders
	F83	Mixed specific skills developmental disorder
	F88	Other psychological developmental disorders
	F89	Unspecified psychological development
**Axis III**		
	F70	Mild mental retardation
	F71	Moderate mental retardation
	F79	Unspecified mental retardation

^a^ICD-10: *International Classification of Diseases, Tenth Revision*.

^b^CAMHS: Child and Adolescent Mental Health Services.

#### Medications

The CAMHS clinic from which the EHR data originated used a diagnostic approach, as shown in Figure 1b, considering the diagnostic categories listed in Table 1.

We used primary keys linking the contact to the recorded diagnosis and medication. Medication information from outside, such as national prescription databases, was not available in the EHR extract. A single contact could contain one or multiple recorded medications, and all recorded medications were retained in the contact-level analysis. As mentioned in the *Introduction* section, medications recorded as prescriptions linked to each contact were based on the ATC classification system [3] and prescribed during the clinical contact with the patient. The presence of an ATC code in this study indicated a prescription or medication documentation event in the CAMHS EHR, but it did not confirm actual medication dispensing, patient adherence, dosage, or treatment duration. For mental health-related diagnoses, ATC nervous system category medications (ie, ATC code with the starting character “N”) [15] were observed to be commonly prescribed in this dataset. Based on suggestions from clinicians, the ATC nervous system category encompasses mostly the psychotropic drugs used in psychiatric treatment. We extracted, preprocessed, and standardized ATC codes to categorize medications, as shown in Table 2, which uses psychotropic medication subcategories [15] and the rest of the nonpsychotropics [16] as a category. The other nonpsychotropic medications shown in Table 2 belong to a ATC nervous system category (N01, N02, N04, and N07), and those with starting ATC code “A” (alimentary tract and metabolism), “B” (blood and blood-forming organs), “C” (cardiovascular systems), “D” (dermatological), “G” (genitourinary system and sex hormones), “H” (systemic hormonal preparation, excluding sex hormones), “J” (anti-infective for systemic use), “M” (musculoskeletal system), “R” (respiratory system), and “S” (sensory organs). Psycholeptics and psychoanaleptics in combination (N06C) as well as antidementia medications (N06D) were not observed to be prescribed in any of the contacts; thus, we did not include these categories. For each contact, we identified medication ATC agent names from ATC code across the Table 2 categories.

**Table 2:** Medication categories by ATC^a^ code prefix.

ATC code	Medication category
N05A	Antipsychotics
N05B	Anxiolytics
N05C	Hypnotics and sedatives
N06A	Antidepressants
N06B	Psychostimulants, agents used for ADHD^b^ and nootropics
N03	Antiepileptics
A, B, C, D, G, H, J, M, N01, N02, N04, N07, R, S	Other nonpsychotropics

^a^ATC: Anatomical Therapeutic Chemical.

^b^ADHD: attention deficit/hyperactivity disorder.

### CAMHS Data Analysis Tool

**Figure 2:**
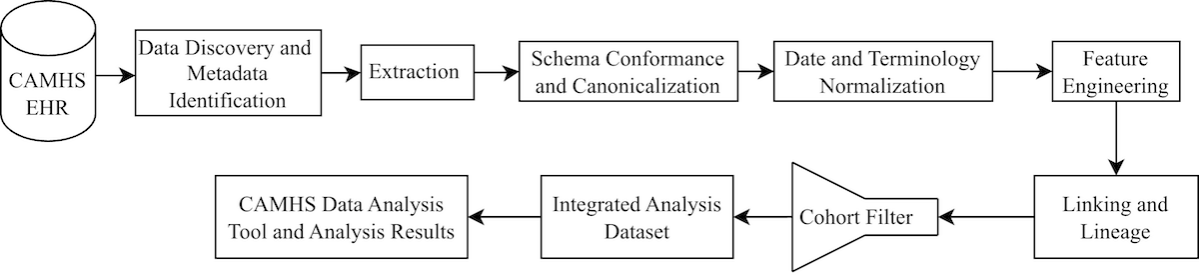
Data pipeline for dataset preparation and analysis. CAMHS: Child and Adolescent Mental Health Services; EHR: electronic health record.

Figure 2 illustrates the data pipeline, which uses rule-based extraction and linkage of patients, episodes, contacts, diagnoses, and medications using a structured schema, and its conformance. The pipeline begins with data discovery and metadata identification from the CAMHS EHR. The useful metadata and attributes are identified and exported as immutable raw extracts to a landing or raw zone within a secure analysis environment. These extracts undergo schema conformance, where data types are enforced, required fields are validated, and columns are renamed to canonical names. This is followed by data normalization, including parsing and standardizing dates, cleaning medication and ATC code terminology, and mapping their descriptions. Next, feature engineering is performed, deriving attributes such as age. Based on the three cohort-filtering conditions, we narrowed the data to the scope of this study. During lineage and linking, the pipeline assembles the medication chain; links patients to episodes and individual contacts; and integrates diagnoses, medications, and contacts to build a complete patient-episode-contact-medication-diagnosis dataset (ie, integrated analysis dataset). This consolidated analysis-ready dataset is consumed by the CAMHS data analysis tool.

Our CAMHS data analysis tool is a web app developed and used to analyze and visualize the data in this study (see Multimedia Appendix 3). We developed and used the tool within a secure analysis environment. The tool was developed using Python, with the libraries *Streamlit* [17] for rapid web app development and *Plotly* [18] for creating interactive visualizations. The tool ingests the preprocessed dataset (ie, integrated analysis dataset) as Parquet files. To ensure data integrity, we mapped data as dictionaries, converting numeric codes (eg, 1 for female and 2 for male and similarly for family relationships and ethnicities). Similarly, the ATC and ICD-10 codes were mapped to corresponding descriptions in human-readable formats (ie, JavaScript Object Notation [JSON] files). We used this tool to obtain axes I, II, and III summaries and analyze patient and contact-level information. The tool offers the following functionality:

- Filtering: Users can filter patient records by ICD-10 codes (axis specific) or ATC codes. Optionally, the age and gender filters are implemented via checkboxes to include or exclude these criteria.
- Patient trajectory visualization: The patient trajectory displays each episode of care, consisting of individual contacts within it, spanning the episode’s duration and including the patient’s age at the start of the episode [19]. Hover annotations display information for each contact, including ICD-10 codes, ATC medication details, and demographic information. The tool supports randomized pagination, ensuring that a manageable subset of patient trajectories is displayed at any one time.
- Interactive visual analytics: Histograms and pie charts illustrate the distribution of demographics, while the patient trajectory provides a detailed view of information at the individual patient and the contact level (eg, medications and diagnoses given per contact).

### Analyses

All analyses were conducted using Python, Structured Query Language, Microsoft Excel, and our CAMHS data analysis tool. The analyses were descriptive and focused on patient contact events as the primary analytic unit. We did not perform inferential modeling or estimate patient-level prevalence from these data. As repeated contacts from the same patient are not independent, the reported percentages should be interpreted as descriptive contact-level proportions rather than inferential estimates. To accomplish this, we first generated a summary for each axis. Sociocultural analysis based on demographic fields was not assessed or interpreted in the study, as substantial missing data in fields, such as parental ethnicity and home language, limited the depth of sociocultural analysis.

### Ethical Considerations

The Individualised Digital DEcision Assist System (IDDEAS) project [20] was assessed by the Regional Committee for Medical and Health Research Ethics (REK Southeast, reference number 2018–2186), which determined that the project fell outside the scope of the Health Research Act and did not require formal ethics approval. Following the REK’s recommendations, the Central Norway Regional Health Authority IT and the local data protection officer approved a risk analysis and data protection impact assessment (DPIA). Informed consent was obtained or waived, and all data were deidentified to ensure participant confidentiality. No compensation was provided, and no identifiable information is present in any images or supplementary materials.

## Results

### Research Question 1: Frequent Diagnoses and Medications in All Contacts

**Figure 3:**
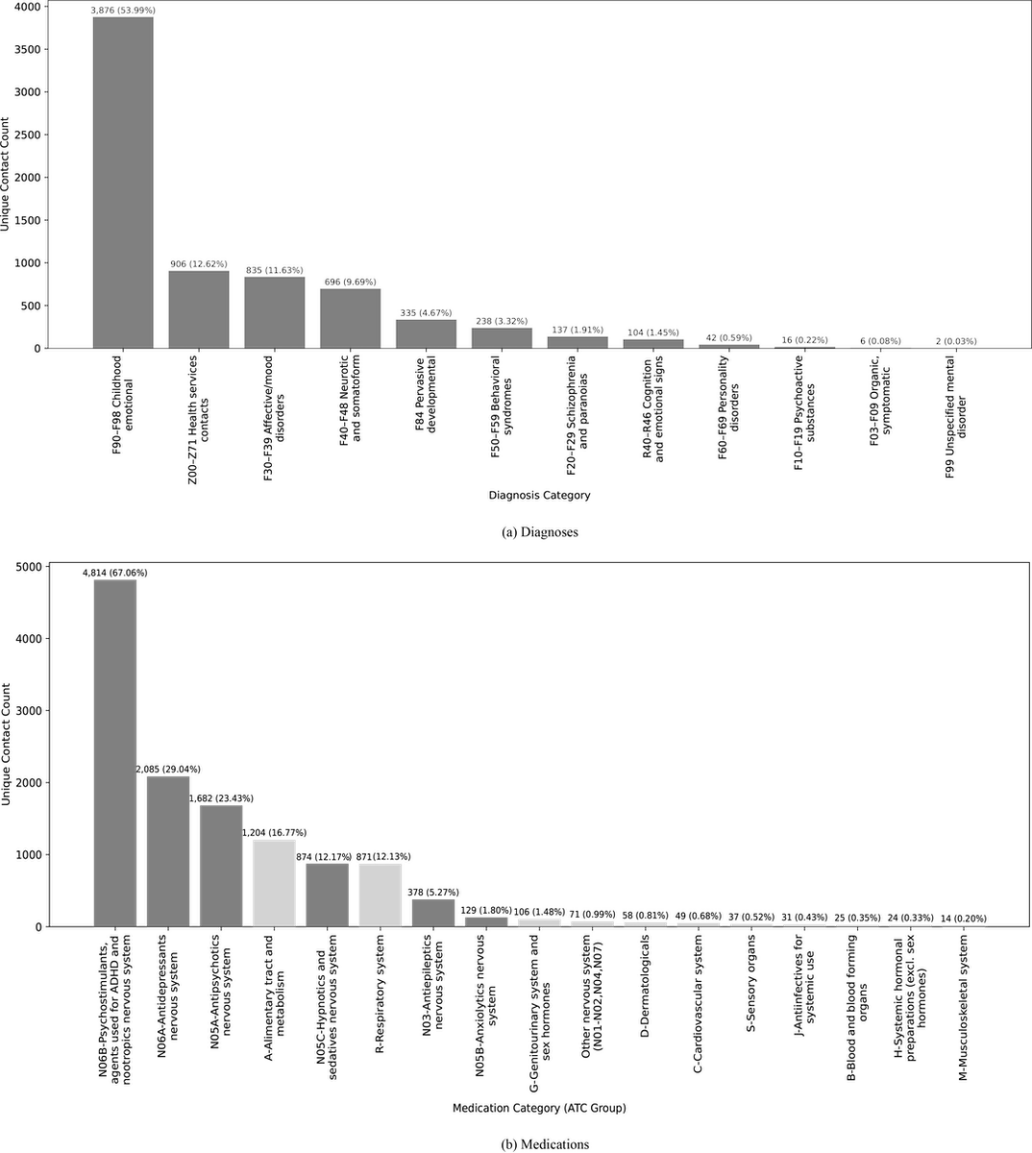
Medication and diagnosis categories in all axis I contacts (n=7179, 99.51%; percentages based on "n"). ADHD: attention deficit/hyperactivity disorder; ATC: Anatomical Therapeutic Chemical.

We calculated and identified the most common primary diagnoses and the most commonly prescribed medications per individual contact, represented in descending order. This was based on the frequency within each corresponding category in axes I, II, and III. The psychotropic medication categories (antipsychotics, anxiolytics, hypnotics and sedatives, antidepressants, psychostimulants, and antiepileptics) are represented by gray bars; other nonpsychotropic medications are represented by light-gray bars (see Figure 3, Multimedia Appendix 4, and Multimedia Appendix 5, respectively).

### Research Question 2: Comorbidities by Diagnosis in Comorbid Contacts

We identified and ranked the frequency of comorbidities across all diagnostic categories on all three axes based on the ratio of comorbid contacts in that diagnosis category to the total comorbid contacts in the axis. The distinction between comorbid and noncomorbid contacts helped pinpoint medication prescriptions for a specific diagnosis. This was necessary because in the EHR data, medication prescriptions are linked to the contact rather than to a specific diagnosis.

### Research Question 3: Medication Patterns by Diagnosis in All Contacts and Excluding Comorbid Contacts

**Figure 4:**
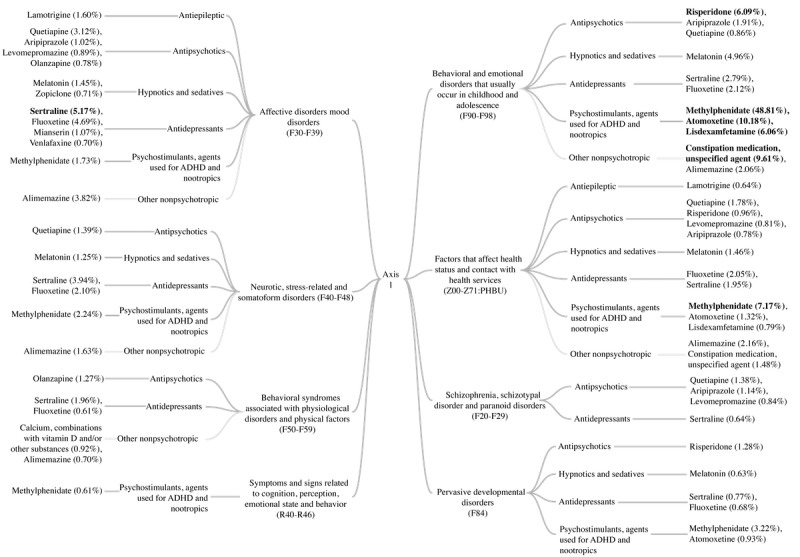
Categorized primary diagnoses and corresponding mapped medications for all axis I contacts (n=7179, 99.51%; percentages based on “n”). A single contact may include multiple mapped medications; therefore, medication percentages represent proportions of all axis I contacts, not prescribing rates within the corresponding diagnosis category. ADHD: attention deficit/hyperactivity disorder.

For each axis, we mapped the intersection of diagnoses and medications. We identified and listed all corresponding medications in each diagnosis category. These results are presented in the form of a mind map (see Figure 4 and Multimedia Appendices 6 and 7) for illustration and clarity. For the other nonpsychotropic medication category, we displayed only the five most frequently prescribed medications to maintain readability. As our focus was on the psychiatric medication categories shown in Table 2, the complete mappings, including proportions, are provided in Multimedia Appendices 6, 7, and 8. Representing the mapping of medications and diagnoses in this way aimed to help the reader understand and navigate which medications were prescribed for which diagnosis categories. The ordering of medications is in descending order, based on the ratio of contacts involving observed medication use to the total number of contacts in the axis.

**Figure 5:**
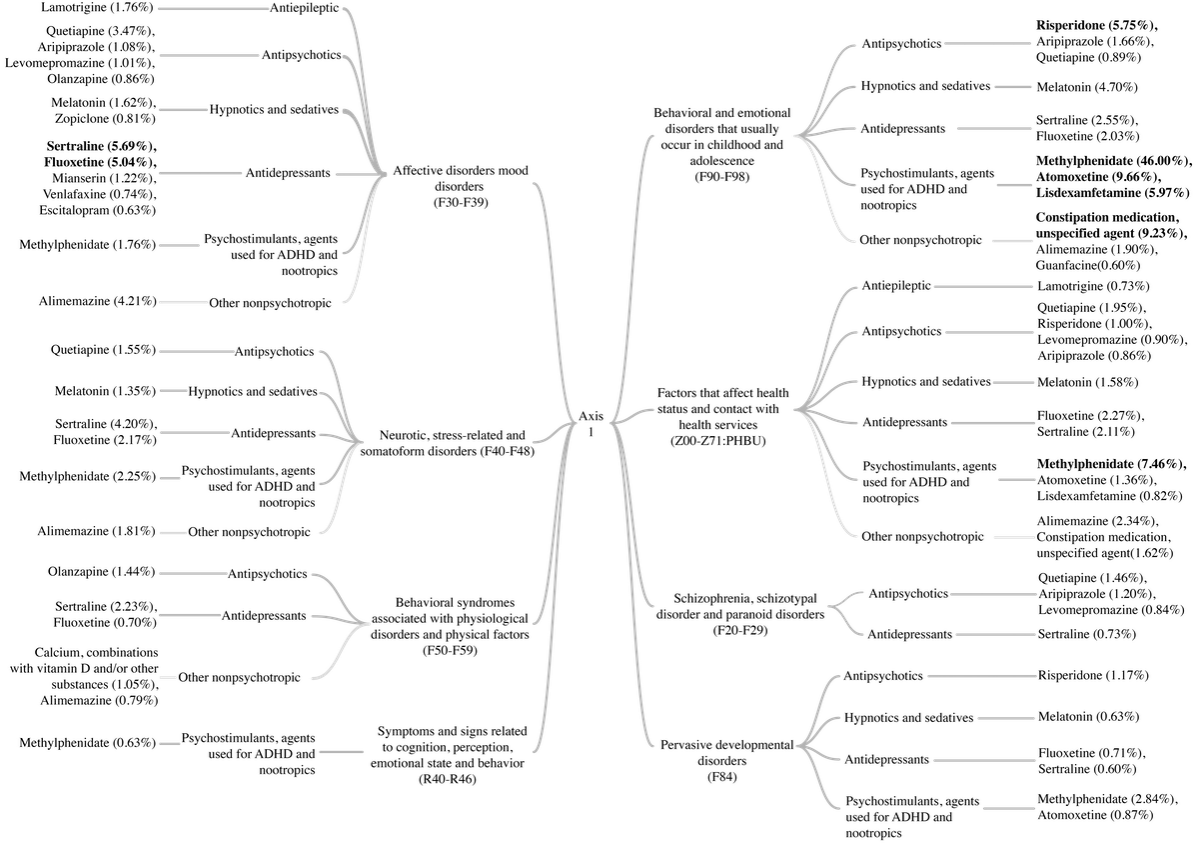
Categorized primary diagnoses and corresponding mapped medications for all noncomorbid axis I contacts (n=6313, 87.94%; percentages based on "n"). A single contact may include multiple mapped medications; therefore, medication percentages represent proportions of all noncomorbid axis I contacts, not prescribing rates within the corresponding diagnosis category. ADHD: attention deficit/hyperactivity disorder.

We separated noncomorbid contacts and examined the intersection of diagnoses and medications across the categories (see Figure 5 and Multimedia Appendices 9 and 10), showing the ratio of noncomorbid contacts involving observed medication use to the total noncomorbid contacts on the axis.

### Characteristics of Patients

Table 3 presents an overview of patients with an axis I diagnosis and their contacts, with analogous summaries for axes II and III provided in Multimedia Appendices 11 and 12, respectively. The analysis involved a total of 7214 contacts, with 7179 (99.51%) classified under axis I, 821 (11.38%) under axis II (Multimedia Appendix 11), and 65 (0.90%) under axis III (Multimedia Appendix 12); the comorbid contacts were classified under multiple axes. The majority of contacts were in axis I. Table 4 lists the diagnostic categories, along with corresponding comorbidity categories observed across axes I, II, and III, arranged in descending order by proportion (see Multimedia Appendix 13). Axis I had a comparatively lower comorbidity ratio: only 12.06% (n=866) of axis I contacts had a comorbid diagnosis. Notably, the comorbidity ratio in axis II was 96.10% (n=789), and axis III had a comorbidity ratio of 96.92% (n=63). The mapped diagnosis medication intersection showed prescribing patterns in all contacts (see Figure 4 and Multimedia Appendices 6 and 7) and those without comorbidity (see Figure 5 and Multimedia Appendices 9 and 10). The noncomorbid contacts included 6313 (87.94%) contacts in axis I, 32 (3.89%) in axis II, and only 2 (3.07%) in axis III.

**Table 3:** Axis I EHR^a^ data overview, excluding diagnosis codes 000, 1000, 1999, 999, X6n, Z724, and F710.^b^

Demographic characteristic		Value
**Cohort**		
	Total patients, N	3410
	Total episodes of care, n	4682
	Total contacts (N=7214), n (%)	7179 (99.51)
**Axis I patient-level contact distribution summary**		
	Contacts per patient, median(IQR; range)	1 (1-2; 1-34)
	Number of patients in top 5% (N=3410), n (%)	171 (5.01)
	Contacts contributed by top 5% (N=7179), n (%)	1474 (20.53)
**Comorbid and noncomorbid contacts (N=7179), n (%)**		
	Total comorbid contacts	866 (12.06)
	Total noncomorbid contacts	6313 (87.94)
**Gender (N=3410), n (%)**		
	Male	1985 (58.21)
	Female	1425 (41.79)
**Episode-of-care start (date)**		
	Oldest	March 31, 1998
	Newest	January 6, 2018
**Episode-of-care end (date)**		
	Oldest	July 9, 1999
	Newest	July 3, 2019
**Age at first episode (years)**		
	Mean (SD)	12 (4)
	Median (IQR; range)	12 (9-15; 1-18)
**Home language (N=3410), n (%)**		
	Not specified	1669 (48.94)
	Norwegian	1681 (49.30)
	Other	36 (1.06)
	Bilingual	24 (0.70)
**Mother’s relation (N=3410), n (%)**		
	Not specified	281 (8.24)
	Biological mother	2916 (85.51)
	Biological father	8 (0.23)
	Adoptive mother	61 (1.79)
	Foster mother	114 (3.34)
	Stepmother	6 (0.18)
	Adoptive father	1 (0.03)
	Foster father	1 (0.03)
	Other	22 (0.65)
**Father’s relation (N=3410), n (%)**		
	Not specified	580 (17.01%)
	Biological father	2569 (75.34)
	Biological mother	25 (0.73)
	Adoptive father	62 (1.82)
	Adoptive mother	2 (0.06)
	Stepfather	62 (1.82)
	Stepmother	1 (0.03)
	Foster father	91 (2.67)
	Foster mother	4 (0.12)
	Other	10 (0.29)
	Spouse/partner	3 (0.09)
**Mother’s ethnicity (N=3410), n (%)**		
	Not specified	1661 (48.71%)
	Norwegian	1675 (49.12)
	Sami	1 (0.03)
	Nordic	9 (0.26)
	European	24 (0.70)
	Asian	13 (0.38)
	African	14 (0.41)
	Latin American	12 (0.35)
	North American	1 (0.03)
**Father’s ethnicity (N=3410), n (%)**		
	Not specified	1758 (51.55)
	Norwegian	1569 (46.01)
	Sami	3 (0.09)
	Nordic	12 (0.35)
	European	33 (0.97)
	Asian	17 (0.50)
	African	11 (0.32)
	Latin American	6 (0.18)
	North American	1 (0.03)
**Total diagnoses and medications**		
	Diagnoses	182
	Medications	118
**Most frequent 5 diagnoses (contact level;** **N=** **7179), n (%)**		
	F900: disturbance of activity or attention	2821 (39.3)
	Z032: observation in case of suspected mental illness or behavioral disorder	818 (11.4)
	F901: hyperkinetic behavior disorder	395 (5.5)
	F321: moderate depressive episode	373 (5.2)
	F845: Asperger’s syndrome	208 (2.9)
**Most frequent 5 medications (contact level;** **N=** **7179), n (%)**		
	N06BA04: methylphenidate	4602 (64.1)
	N06AB06: sertraline	1278 (17.8)
	N06BA09: atomoxetine	940 (13.1)
	N06AB03: fluoxetine	919 (12.8)
	A06BA04: constipation medication, unspecified agent	861 (12.0)

^a^EHR: electronic health record.

^b^Demographic percentages are based on the total patient count (N). Percentages for the five most frequent diagnoses and medications are based on the total number of axis I contacts (n=7179, 99.51%) as the denominator.

**Table 4:** Comorbid diagnosis categories in all three axes (showing only with proportion ≥0.6%).^a^

Axis and primary categories			Rank 1 (%)	Rank 2 (%)		Rank 3 (%)		Rank 4 (%)		Rank 5 (%)		Rank 6 (%)
**Axis I (n=866 comorbid contacts)**												
	F20-F29 (schizophrenia and paranoia)	Axis III: F70 (0.69)		—^b^	—		—		—		—	
	F30-F39 (affective/mood disorders)	Axis II: F81 (1.85)		Axis I: F30-F39 (1.15)	Axis II: F80 (0.69)		—		—		—	
	F40-F48 (neurotic and somatoform disorders)	Axis II: F81 (3.12)		Axis II: F83 (0.69)	—		—		—		—	
	F84 (pervasive developmental disorders)	Axis II: F81 (3.81)		Axis II: F83 (1.62)	Axis II: F80 (1.27)		Axis II: F82 (0.81)		—		—	
	F90-F98 (childhood emotional disorders)	Axis II: F81 (41.11)		Axis II: F80 (15.24)	Axis II: F83 (8.20)		Axis II: F82 (4.97)		Axis III: F70 (4.27)		Axis I: F90-F98 (3.46)	
	Z00-Z71 (health services contacts)	Axis II: F81 (3.35)		Axis II: F80 (1.27)	Axis I: F90-F98 (0.81)		—		—		—	
**Axis II (n=789 comorbid contacts)**												
	F80 (speech and language)	Axis I: F90-F98 (16.73)		Axis III: F70 (1.39)	Axis I: F84 (1.39)		Axis I: Z00-Z71 (1.39)		Axis I: F30-F39 (0.76)		Axis I: F40-F48 (0.63)	
	F81 (school skills and learning difficulties)	Axis I: F90-F98 (45.12)		Axis I: F84 (4.18)	Axis I: Z00-Z71 (3.68)		Axis I: F40-F48 (3.42)		Axis I: F30-F39 (2.03)		—	
	F82 (motor skills disorders)	Axis I: F90-F98 (5.45)		Axis I: F84 (0.89)	Axis I: Z00-Z71 (0.63)		—		—		—	
	F83 (mixed specific skills development disorder)	Axis I: F90-F98 (9.00)		Axis I: F84 (1.77)	Axis I: F40-F48 (0.76)		—		—		—	
**Axis III (n=63 comorbid contacts)**												
	F70 (mild mental retardation)	Axis I: F90-F98 (58.73)		Axis II: F80 (17.46)	Axis I: F20-F29 (9.52)		Axis I: F84 (7.94)		Axis I: Z00-Z71 (4.76), Axis II: F82 (4.76)		Axis I: F30-F39 (1.59) Axis II: F81 (1.59) Axis II: F83 (1.59)	
	F71 (moderate mental retardation)	Axis I: F84 (3.17)		Axis I: F90-F98 (3.17)	Axis I: F20-F29 (1.59)		—		—		—	
	F79 (unspecified mental retardation)	Axis I: F90-F98 (3.17)		—	—		—		—		—	

^a^Percentages are at the contact level within clinical episodes. Comorbidities in the same category represent two distinct *International Classification of Diseases, Tenth Revision* (ICD-10) codes within the same primary category block.

^b^Not applicable.

#### Axis I: Clinical Psychiatric Syndrome

As shown in Figure 3a, axis I (n=7179, 99.51%) contacts exhibited childhood behavioral-emotional disorders (ICD-10 F900 [disturbance of activity] and F901 [hyperkinetic disorder]) as the most frequent disorders (F90-F98; n=3876, 53.99%), followed by health service contacts, potentially representing consecutive visits or follow-ups (Z codes; n=906, 12.62%), affective mood disorders (F30-F39; n=835, 11.63%), and neurotic disorders (F40-F48; n=696, 9.69%). Consistent with these diagnostic categories, psychostimulant medications (N06B; n=4814, 67.06%), followed by antidepressants (N06A; n=2085, 29.04%), were the most observed medications (Figure 3b). Table 4 shows that among the 866 (12.06%) comorbid contacts in axis I, in 41.11% (n=356), childhood behavioral emotional disorders (F90-F98) were comorbid with specific learning disorders (F81), followed by speech/language disorder (F80; n=132; 15.24%), mixed specific skills development disorder (F83; n=71, 8.2%).

Figures 4 and 5 show the axis I diagnoses and mapped prescribed medications (see Multimedia Appendix 8), considering all contacts (Figure 4) and only noncomorbid contacts (Figure 5). Percentages are based on the total contact denominator used in the corresponding figure and should not be interpreted as within-diagnosis prescribing rates. For example, as shown in Figure 4 for childhood behavioral-emotional disorders (F90-F98), the most frequent medication was methylphenidate, observed in 48.81% (n=3504) of all contacts in axis I, followed by atomoxetine (n=731, 10.18%) and risperidone (n=435, 6.06%). As medication code A06BA04 could not be reliably mapped to a specific generic substance, and standard constipation drugs are classified under A06A, this medication was therefore labeled as “Constipation medications, unspecified agent.” For affective disorders and mood disorders (F30-F39), sertraline (n=371, 5.17%) and fluoxetine (n=337, 4.69%) were frequent. For neurotic, stress-related, and somatoform disorders (F40-F48) also, sertraline (n=283, 3.94%) and fluoxetine (n=151, 2.10%) were frequent.

Figure 5 shows medications prescribed to noncomorbid contacts only. For example, in the absence of comorbidity for the same F90-F98 diagnoses category, methylphenidate was observed in 46% (n=2904) of axis I noncomorbid contacts, followed by atomoxetine (n=610, 9.66%), and lisdexamfetamine (n=377, 5.97%). For affective disorders and mood disorders (F30-F39), the antidepressants sertraline (n=359, 5.69%) and fluoxetine (n=318, 5.04%) were frequent. For neurotic, stress-related, and somatoform disorders (F40-F48) also, sertraline (n=265, 4.2%) and fluoxetine (n=137, 2.17%) were frequent.

#### Axis II: Specific Developmental Disorders

Axis II contacts (n=821, 11.38%) had specific developmental disorders of scholastic skill (F81; n=481, 58.59%), which in ICD-10 also include specific reading disorders and learning difficulties as the most common diagnosis, followed by speech/language issues (F80; n=173, 21.07%), mixed disorders (F83; n=102, 12.42%), and motor disorders (F82; n=59, 7.19%); see Multimedia Appendix 4. Axis II had lower medication exposure but substantial comorbidity with axis I. Among medications, overwhelmingly, psychostimulants (n=724, 88.19%), followed by antidepressants (n=138, 16.81%), were observed (see Multimedia Appendix 4). Other medication categories, such as alimentary tract and metabolism (n=127, 15.47%), included constipation medications and were often observed across all axes. The axis II comorbidities (n=789, 96.10%) shown in Table 4 also revealed that childhood behavioral-emotional disorders (F90-F98) were mostly comorbid with school skills and learning difficulties (F81; n=356, 45.12%), speech/language issues (F80; n=132, 16.73%), mixed specific skills development disorders (F83; n=71, 9.00%), and motor skills disorders (F82; n=43, 5.45%). Multimedia Appendices 6 and 9 show the mapped medications prescribed for axis II diagnosis categories, considering all contacts (Multimedia Appendix 6) and only noncomorbid contacts (Multimedia Appendix 9). They show the predominance of psychostimulant medications across diagnostic categories.

#### Axis III: Mental Developmental Disabilities

Axis III had only 65 (0.90%) contacts with mild, moderate, or unspecified retardation. The diagnosis was dominated by mild retardation (F70; see Multimedia Appendix 5), which in ICD-10 includes F700-F701 (mild behavioral retardation). The diagnosis also showed frequent comorbidity with, especially, childhood behavioral-emotional disorders (F90-F98; n=37, 58.73%) and other neurodevelopmental conditions (see Table 4). With 96.92% (n=63) comorbidity, axis III showed the use of antipsychotic and psychostimulant medications (see Multimedia Appendix 5). Axis III diagnoses and corresponding medication mapping (see Multimedia Appendices 7 and 10) showed that methylphenidate among psychostimulants and risperidone among antipsychotics were common; this is an observation of the small number of contacts in axis III, not a disorder-specific treatment pattern for intellectual disability in isolation or for epidemiological interpretation.

## Discussion

### Principal Findings

CAMHS EHR data extraction and preprocessing are complex because patient, episode-of-care, and contact-level records are integrated [4]. We extracted, transformed, and mapped 25 years of longitudinal CAMHS EHR data into a singular contact-level dataset, enabling multilevel analyses of patient demographics, diagnostic and comorbidity patterns, medication-prescribing patterns, and their interrelationships. To the best of our knowledge, this is the first study to extract, transform, mine, and map 25 years of CAMHS EHR data into a contact-level dataset for analyses of diagnostic and medication-prescribing patterns.

Using percentages for axis-specific contacts rather than patient-level prevalence estimates, we observed that among medicated contacts in specialist CAMHS, axis I clinical psychiatric syndromes were most common, particularly childhood behavioral-emotional disorders (F90-F98), which also consist of ADHD (53.99%), health service contacts (Z codes) representing follow-up contacts (12.62%), and affective mood disorders (11.63%). These contacts were dominated by psychostimulant medications, followed by antidepressants, usually corresponding to the treatment of ADHD and mood disorders [6,21]. The presence of risperidone, a second-generation antipsychotic, reflects targeted short-term use for severe behavioral dysregulation [22]. In noncomorbid axis I contacts, rates of methylphenidate remained high at 46.0% for childhood behavioral-emotional disorders (F90-F98), while selective serotonin reuptake inhibitor antidepressant (sertraline, 2.55%; fluoxetine, 2.03%), antipsychotics (risperidone, 5.75%; aripiprazole, 1.66%), hypnotics (melatonin, 4.70%), and nonpsychotropic medication (constipation medications: unspecified agent, 9.23%; alimemazine, 1.90%) were more evenly distributed, indicating more diversified pharmacotherapy when no secondary diagnoses are present. In addition, of the 866 comorbid contacts in axis I, for childhood behavioral-emotional disorders (F90-F98), 41.11% of contacts were also comorbid with specific developmental disorders (F81), highlighting the potential benefits of screening children presenting with childhood behavioral-emotional disorders or ADHD symptoms for developmental disorders.

In axis II (developmental disorders), school skills and learning difficulties (58.59%) were the most frequently occurring diagnoses, followed by speech/language issues (21.07%), mixed specific skills development disorder (12.42%), and motor disorders (7.19%). The high comorbidity between axis I and II, as well as the use of psychostimulants and antidepressants, also points to the medications being prescribed for childhood behavioral-emotional disorder comorbidities, with melatonin and risperidone to address sleep disturbances and behavioral challenges [23]. The axis II noncomorbid contacts, which exhibit a decrease in psychostimulant use and a relative increase in nonpsychostimulant use, reflect more diversified pharmacotherapy and some monotherapy patterns when no secondary diagnoses were present.

Axis III contacts overwhelmingly involved mild intellectual disability (84.62%) and almost always included comorbid diagnoses. Accordingly, the observed methylphenidate and risperidone use in axis III contacts should not be interpreted as evidence of efficacy for intellectual disability itself [24,25]. Rather, these medications were recorded in highly comorbid contacts in which the prescribing indication was likely related to co-occurring behavioral or psychiatric symptoms.

The high comorbidity rates of axis II and axis III contacts with axis I contacts indicate how clinicians choose and use medications when managing patients with multiple comorbid conditions, and the medications may be prescribed to treat the comorbid diagnoses. Axes II and III exhibit a particularly pervasive overlap between neurodevelopmental and intellectual disabilities and childhood behavioral-emotional disorders (F90-F98), leading to frequent symptomatic methylphenidate/atomoxetine and risperidone use. The axis I cohort exhibiting lower comorbidity than axes II and III likely reflects the WHO multiaxial classification structure, which indicates that axis II and III codes capture long-term developmental or intellectual disorders commonly recorded alongside axis I behavioral and clinical conditions. Often, the axis I conditions are linked to medication use for treatment. This indicates that comorbid behavioral disorder likely drives clinical management and prescribing decisions, even when the intellectual disability is coded as the primary diagnosis.

The use of psychostimulants, antidepressants, hypnotics and sedatives, antipsychotics, and other medication categories in CAMHS indicates multifaceted pharmacological management in routine clinical practice settings. This highlights the need for integrated neurodevelopmental screening; coordinated care, including psychosocial interventions; and informed resource allocation to improve long-term prognosis [26]. The predominance of psychoanaleptic (N06A, N06B) medications underscores that stimulant and antidepressant therapies must be tailored alongside interventions for comorbid learning and speech disorders to optimize outcomes [27]. Medication-prescribing patterns characterized by psychostimulants for ADHD and antipsychotics for behavioral disorders broadly align with clinical practice but may raise concerns about the use of antipsychotics in individuals with mild and moderate intellectual disabilities [7,28]. These findings emphasize the importance of careful medication management, offering important implications for future clinical practice and policy enhancements. Policy initiatives should strengthen oversight and access to supportive therapies, and future research will evaluate long-term outcomes and refine evidence-based prescribing strategies to optimize safety and efficacy in these complex patient groups.

Medication approval dates in Norway (see all approval dates in Multimedia Appendix 14) provide a historical context for the available agents across the study period. For example, in the psychostimulants category, methylphenidate was mainly prescribed, followed by atomoxetine. The reason could be that although immediate-release methylphenidate was first approved in Norway in 1956 (see Multimedia Appendix 14) and extended-release methylphenidate was approved for use in Norway in 2002 [29], atomoxetine was not approved until 2004, and our data are from 1995. Therefore, the medication’s approval date and release in Norway may have influenced its usage proportion. However, we did not formally model time trends, segmented changes, or policy effects across the 25-year window. Therefore, differences in observed medication frequencies should not be causally attributed to approval dates, organizational reforms, or guideline changes in this study. Future work should analyze prescribing patterns longitudinally by calendar period. The prescription of particular medications within each medication category also may have been influenced by the Norwegian government, CAMHS clinic policies, individual CAMHS physician preferences, or the preferences of other health care personnel. In addition, depending on the EHR system and the updates made to it over the years, certain information may not have been recorded.

Given that prescribing should follow evidence-based prescribing guidelines, the use of antipsychotics for intellectual disability especially may further indicate the need for monitoring, clear protocols, and integrated multidisciplinary care that enhance psychosocial and nonpharmacological interventions [30]. Policy efforts should focus on providing clear guidelines and monitoring frameworks for psychotropic use in developmental and intellectual disabilities, as well as ensuring access to nonpharmacological interventions, such as psychosocial first line of treatment, including behavioral therapy, educational, and speech support. The use of nonpharmacological interventions is evident, as we observed that of all of the 41,411 patient contacts, only 22.17% had a medication code, indicating that medication was not always the first and foremost choice. This could be potentially further explored from the text notes, behavioral narratives, or patient-reported outcomes.

### Strengths and Limitations

The strength of our study lies in mapping a large-scale, long-term, real-world EHR at the individual contact level, visualizing the most fine-grained encounters involving diagnoses and medication prescriptions. We combined patient, episode, and contact-level information to provide longitudinal insights into diagnostic and prescribing patterns. We applied a systematic WHO multiaxial diagnosis and ATC medication classification, used by the Norwegian CAMHS, to identify comorbid diagnoses and prescribed medications. All results were categorized using the same classification system and summarized in tables and graphics to enhance readability and accessibility. We acknowledge that the ICD-10 and multiaxial diagnosis axis classification provides a useful taxonomy; however, in clinical practice, these categories often overlap, and coding precision and practices may vary, meaning that the contact level may not always reflect clear-cut clinical distinctions.

We note that the medication codes in the EHR represent prescription events and do not confirm actual dispensing, patient adherence, or dosage; they serve, instead, as proxies for clinical treatment intention. The analysis is descriptive rather than inferential; repeated contacts from the same patient are not statistically independent. We acknowledge the following as potential sources of bias in this study: contact-based analysis, medicated-only sampling, missing demographic data, omission of axes IV-VI, unstructured data, and the small size of the axis III sample. As this was a descriptive study, we made these biases transparent and constrained our interpretation accordingly. Future studies could refine prescribing stratification, including contacts without medication prescription, analyze prescriptions based on progression of a disease over time, apply standardized comorbidity indices, focus on polypharmacy scores, and evaluate nonpharmacological alternatives to reduce the psychotropic burden in complex CAMHS populations. The analysis tool is a proof of concept designed to extract and visualize data from our specific EHR. However, it requires external validation in other EHR systems before it can be used elsewhere. As this study was descriptive and limited to contacts with medication prescriptions, conclusions about guideline adherence are not drawn.

### Conclusion

This study demonstrated an analytic pipeline that enabled the mining and mapping of Norwegian CAMHS EHRs over a 25-year period, allowing analysis of diagnostic patterns, comorbidities, and medication-prescribing practices across axes I-III at the individual contact level. We observed that CAMHS prioritize psychosocial interventions, but among the medicated patients, they mostly encounter axis I diagnoses, mostly childhood behavioral-emotional disorders. The axis I diagnoses are predominantly linked to medication use, with axis II and III diagnoses frequently observed to be comorbid with axis I. This study presents a proof of concept in which extensive EHR data collected over decades can be mined and mapped, allowing users to transform complex, multiaxial/multidimensional records into interpretable formats for various purposes, such as understanding clinical decision-making, managing resource allocation, planning, and developing policies. Our methods transform EHRs from “black boxes” into readily accessible information that can be used to inform and improve clinical practice, service use, and ultimately patient outcomes.

## Appendix

Multimedia Appendix 1 Dataset variables and their brief descriptions.

Multimedia Appendix 2 Schema of the dataset variables and tables used from the EHR database.

Multimedia Appendix 3 CAMHS data analysis tool snapshots.

Multimedia Appendix 4 Frequency of diagnoses using ICD-10 categories and frequency of prescribed medications using ATC categories in all axis II 821 contacts.

Multimedia Appendix 5 Frequency of diagnoses using ICD-10 categories and frequency of prescribed medications using ATC categories in all axis III 65 contacts.

Multimedia Appendix 6 Mapping of diagnoses and the corresponding medications for all 821 contacts in axis II.

Multimedia Appendix 7 Mapping of diagnoses and corresponding medications for all 65 contacts in axis III.

Multimedia Appendix 8 Complete axis I diagnosis and medications mapping for all contacts, and for non-comorbid contacts only.

Multimedia Appendix 9 Mapping of diagnoses and corresponding medications for the 32 non-comorbid contacts in axis II.

Multimedia Appendix 10 Mapping of diagnoses and corresponding medications for the 2 non-comorbid contacts in axis III.

Multimedia Appendix 11 Axis II EHR data overview, filtered based on age between 0 ≤ 19, with episode of care from 1995 onwards, medicated, and excluding diagnosis codes 2000, 2999, 999, 000. Percentages for the 5 most frequent diagnoses and medications are based on the total number of axis II contacts (C2) = 821 as the denominator.

Multimedia Appendix 12 Axis III EHR data overview, filtered based on age between 0 ≤ 19, with episode of care from 1995 onwards, medicated, and excluding diagnosis codes 3000, 3999, 1, 2, 3, 4, 5, 9, 99. Percentages for the 5 most frequent diagnoses and medications are based on the total number of axis III contacts (C3) = 65 as the denominator.

Multimedia Appendix 13 Complete table showing all comorbid diagnoses along with the proportions across all three axes.

Multimedia Appendix 14 Frequently used medications in each ATC category across all three axes, with their approval date in Norway.

## Abbreviations

ADHD: attention deficit/hyperactivity disorder
ATC: Anatomical Therapeutic Chemical
CAMHS: Child and Adolescent Mental Health Services
CGAS: Children’s Global Assessment Scale
EHR: electronic health record
ICD-10: *International Classification of Diseases, Tenth Revision*
WHO: World Health Organization

## Data Availability

This study used the *barne-og ungdomspsykiatri* data EHR managed by St Olavs Hospital in Trondheim, Norway. Due to the sensitive nature of the data, they cannot be made publicly available with the study. To request access, please contact Research Department, St Olavs Hospital, Trondheim, Norway. The applicant must comply with any requirements or guidelines provided by the head of the project, as well as any nondisclosure agreements, the Regional Committee for Medical and Health Research Ethics, the Data Access Committee, and the Helseundersøkelsen i Trøndelag Cloud. Breach of legal or formal requirements is punishable under Norwegian law. The code is available online [31].
