# Supplementary material for: Mining and Mapping 25 Years of Medication Use in Child and Adolescent Mental Health Services: Contact-Level Descriptive Analysis of Electronic Health Records
Source: JMIR Med Inform. 2026 Jun 16;14:e86066. doi: 10.2196/86066 (PMC13320007; doi:10.2196/86066)
Supplement: Multimedia Appendix 1 [file medinform_v14i1e86066_app1.pdf]

**Column name**	**Description**
patient (pasientnr)	Unique patient identifier.
episode_of_care (saknr)	Identifier for the care episode.
gender (kjonn)	Patient's gender.
age (combined use of fdt, henvdato, igangdato)	Patient age at episode start, calculated from birth & referral dates.
episode_start (igangdato)	Date the case was opened.
episode_end (avsldato)	Date the case was closed.
atc_code (atckode)	ATC medication code at contact.
atc_name (atcnavn)	ATC medication name at contact.
diagnosis_code (diagnose)	ICD-10 code recorded at contact.
diagnosis_axis (akse)	Axis/category of the diagnosis.
primary_diagnosis (hoved)	Main diagnosis at contact.
diagnosis_age (use of fdt, diag_dato)	Age when diagnosed, calculated from birth & diagnosis dates during contact.
diag_date (diag_dato)	Date of diagnosis at contact.
contact (opphold_id)	Identifier for the patient stay/contact.
contact_start (opphold_start_date)	Start date of the stay/contact.
contact_end (opphold_end_date)	End date of the stay/contact.
