# Supplementary figures and images for: Mining and Mapping 25 Years of Medication Use in Child and Adolescent Mental Health Services: Contact-Level Descriptive Analysis of Electronic Health Records
Source: JMIR Med Inform. 2026 Jun 16;14:e86066. doi: 10.2196/86066 (PMC13320007; doi:10.2196/86066)

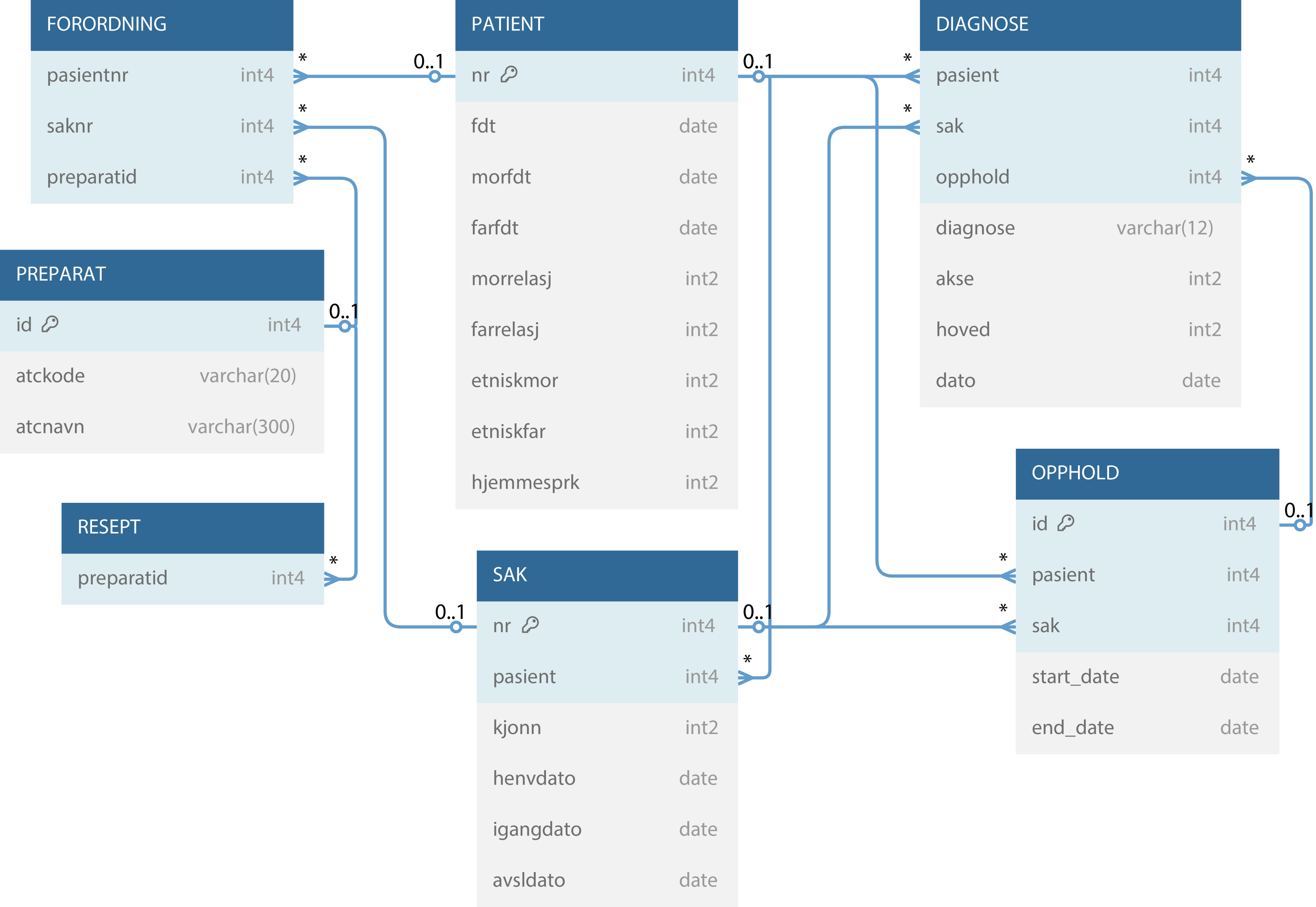

Supplement: Multimedia Appendix 2 [file medinform_v14i1e86066_app2.pdf]

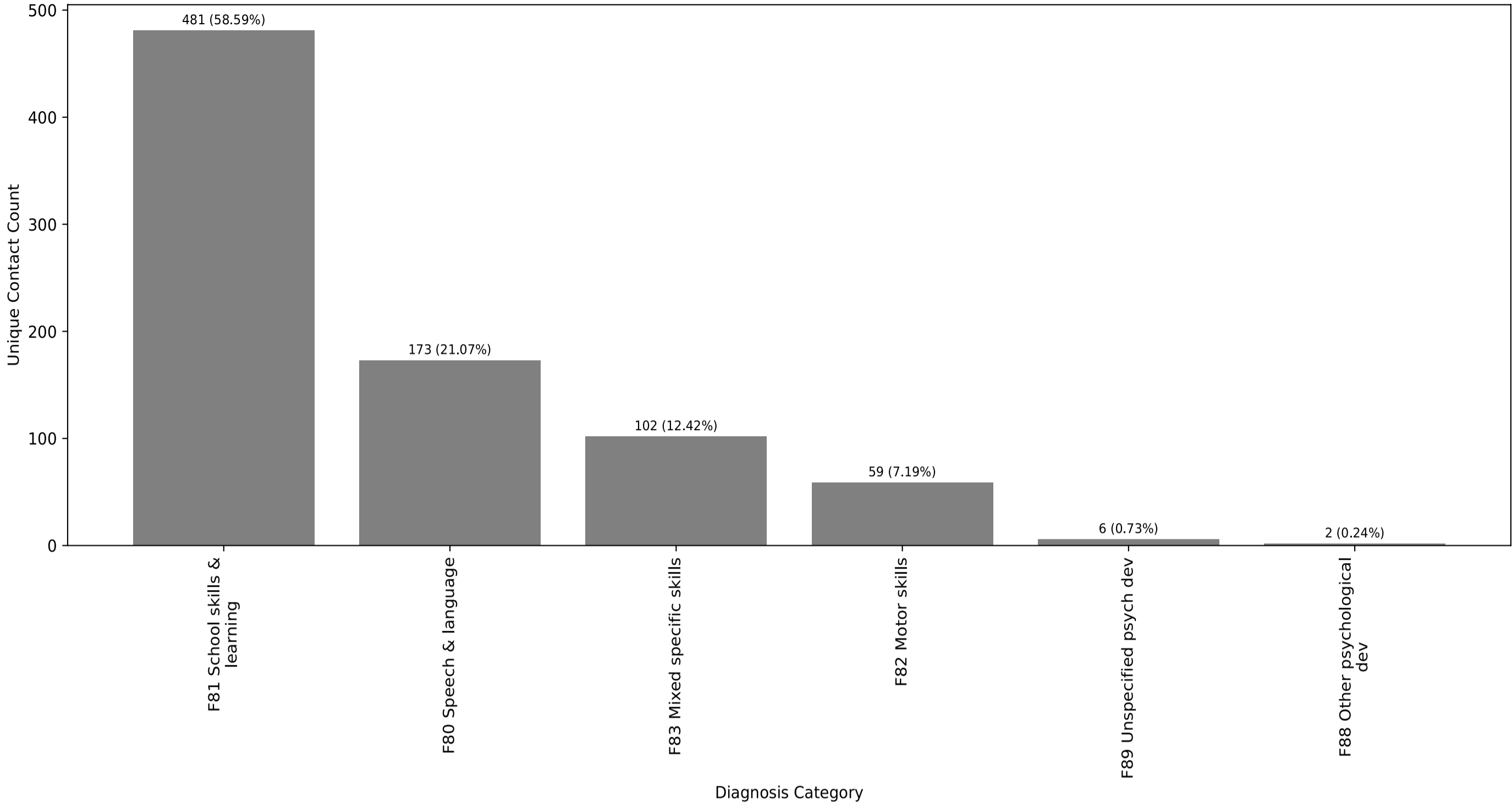

(a) Diagnoses

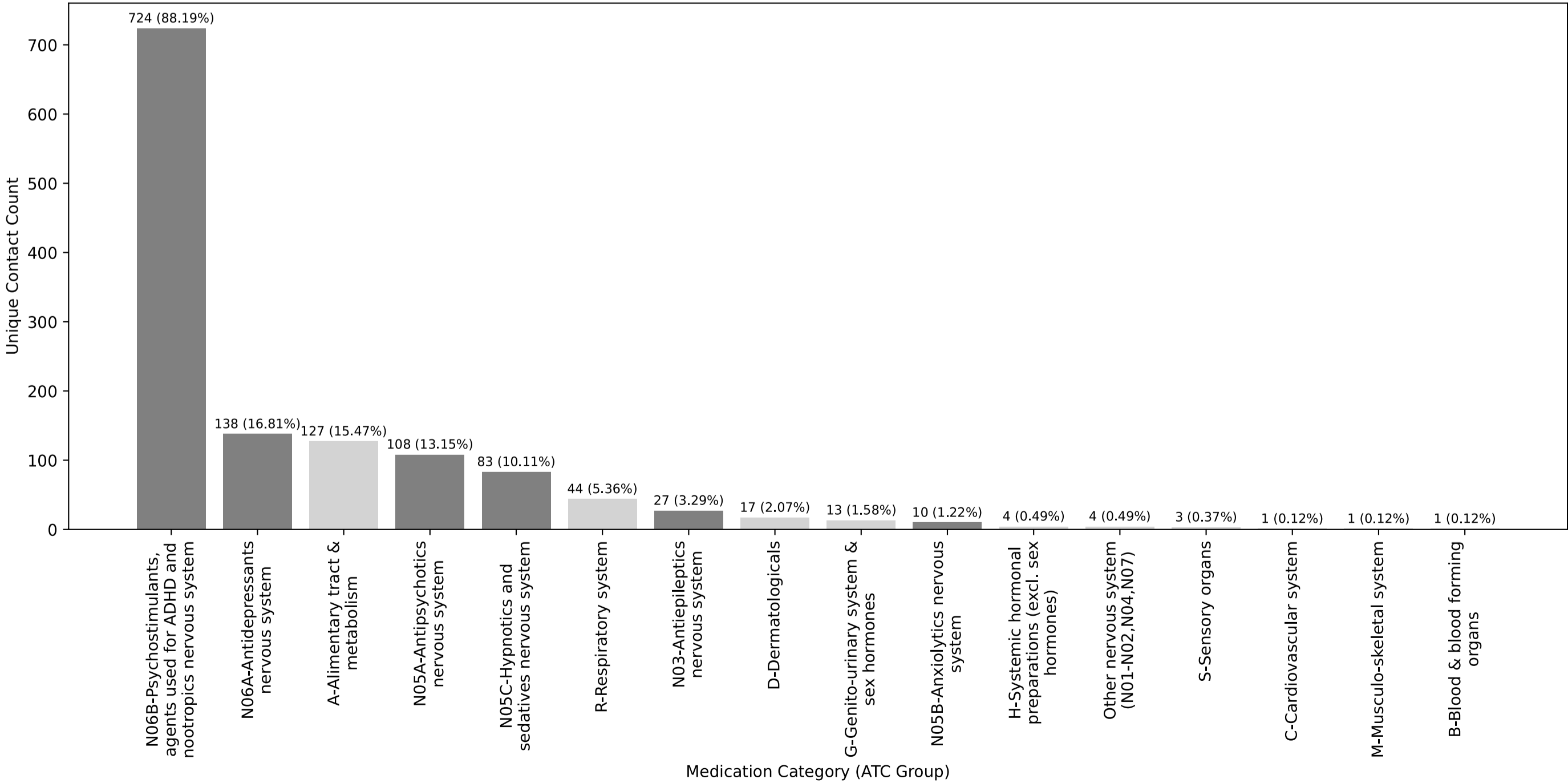

(b) Medications

Supplement: Multimedia Appendix 4 [file medinform_v14i1e86066_app4.pdf]

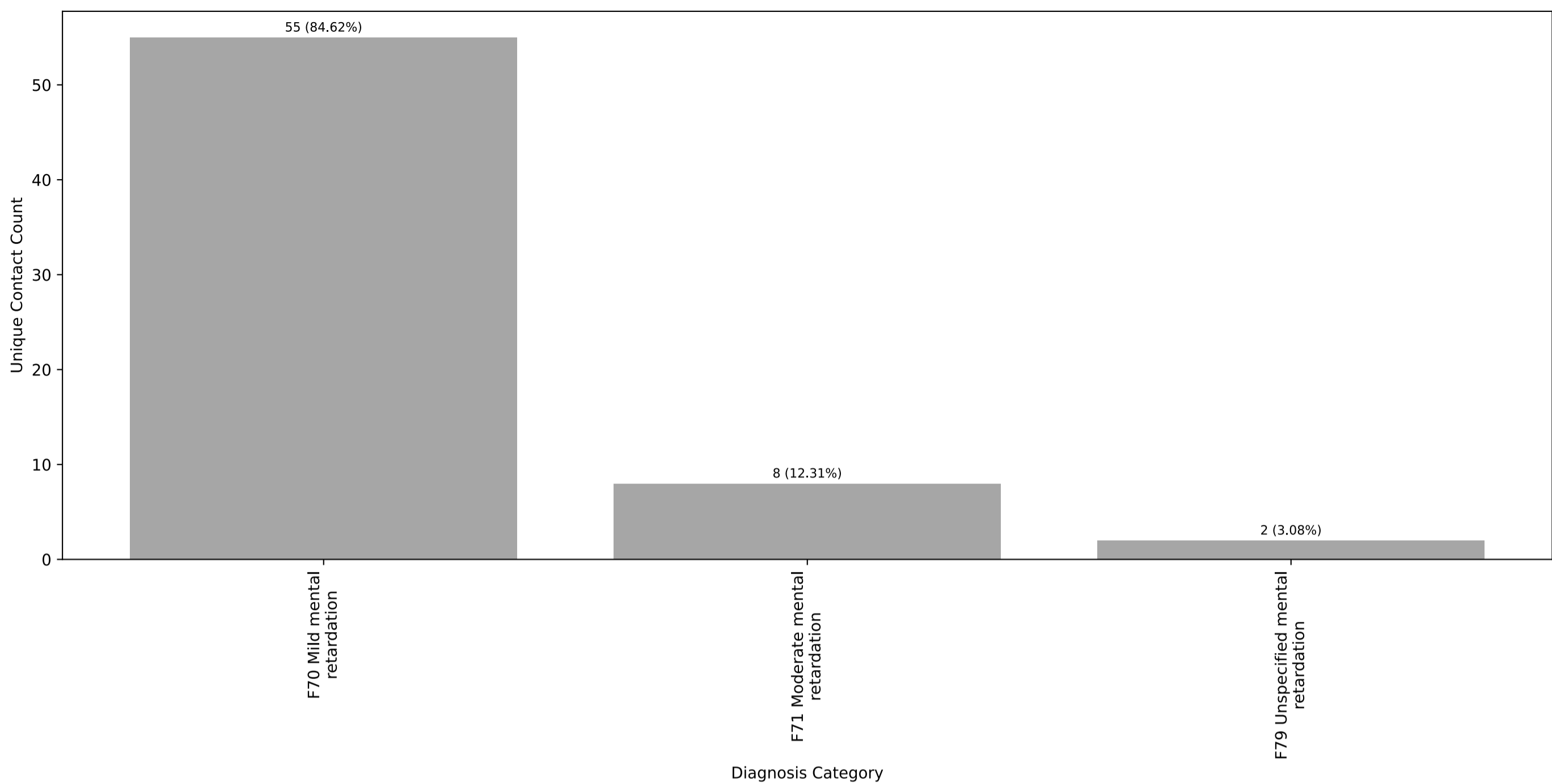

(a) Diagnoses

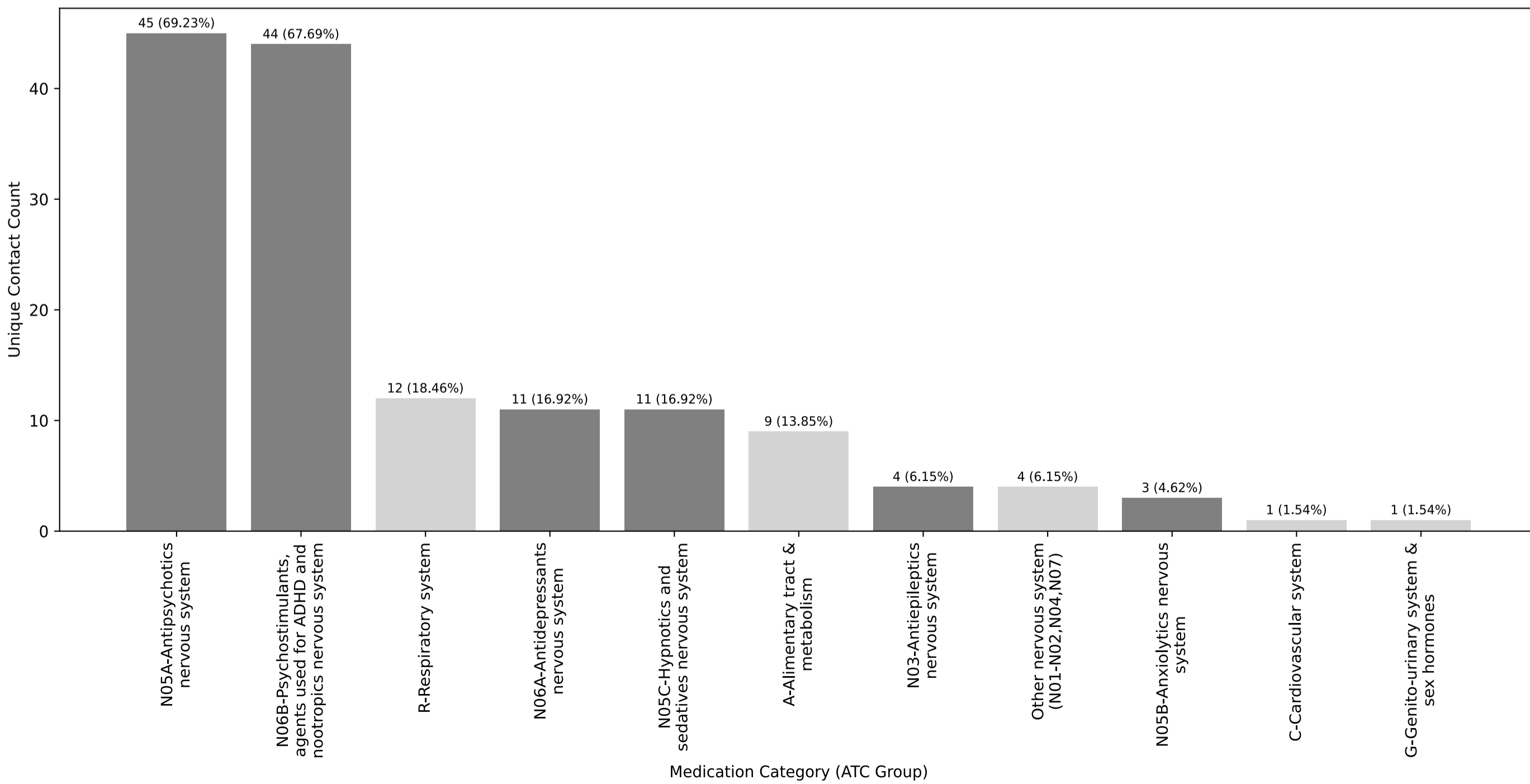

(b) Medications

Supplement: Multimedia Appendix 5 [file medinform_v14i1e86066_app5.pdf]

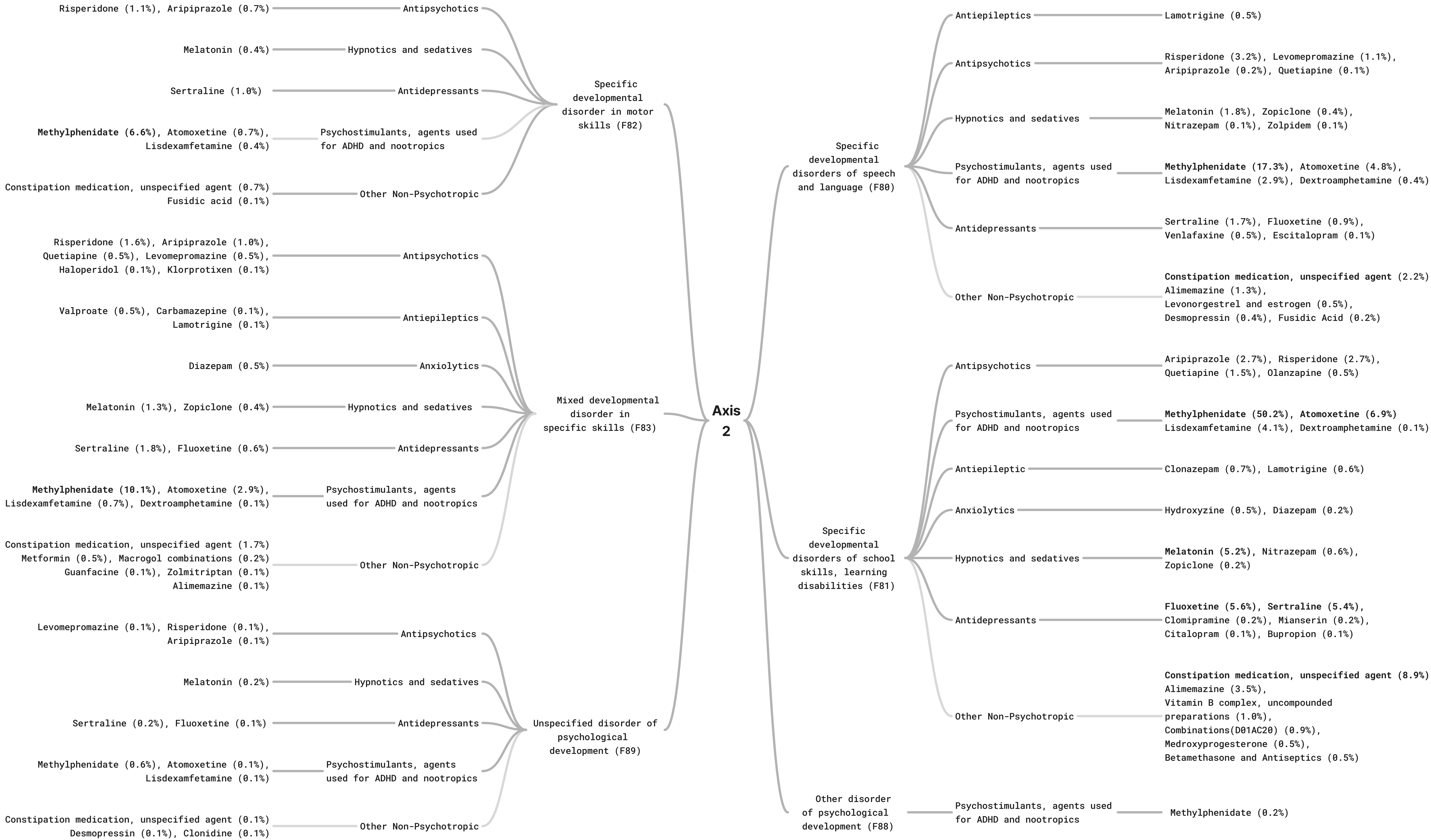

Supplement: Multimedia Appendix 6 [file medinform_v14i1e86066_app6.pdf]

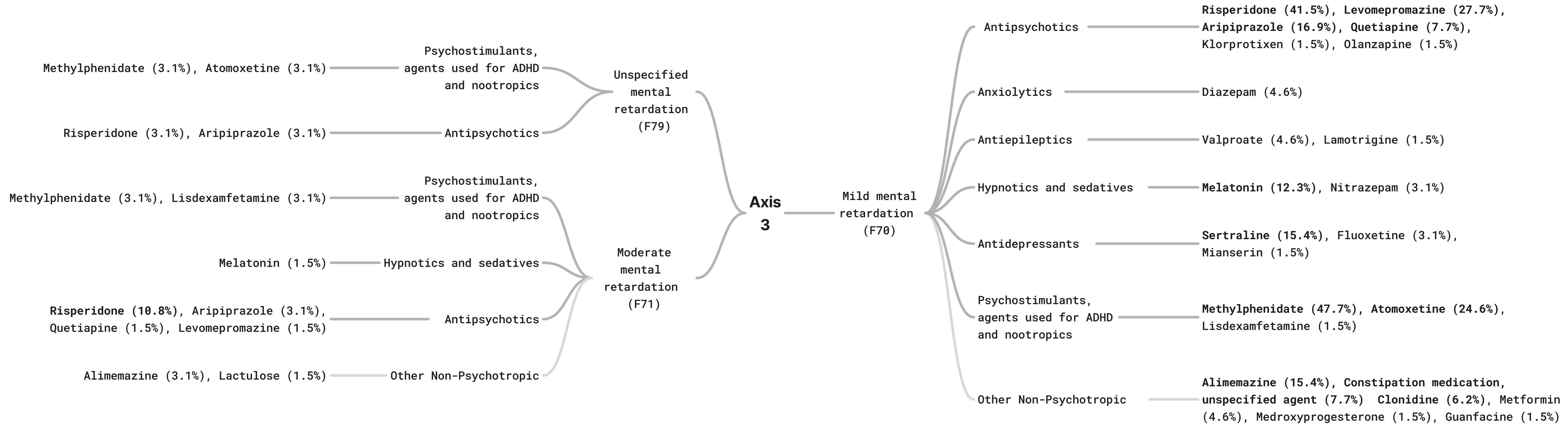

Supplement: Multimedia Appendix 7 [file medinform_v14i1e86066_app7.pdf]

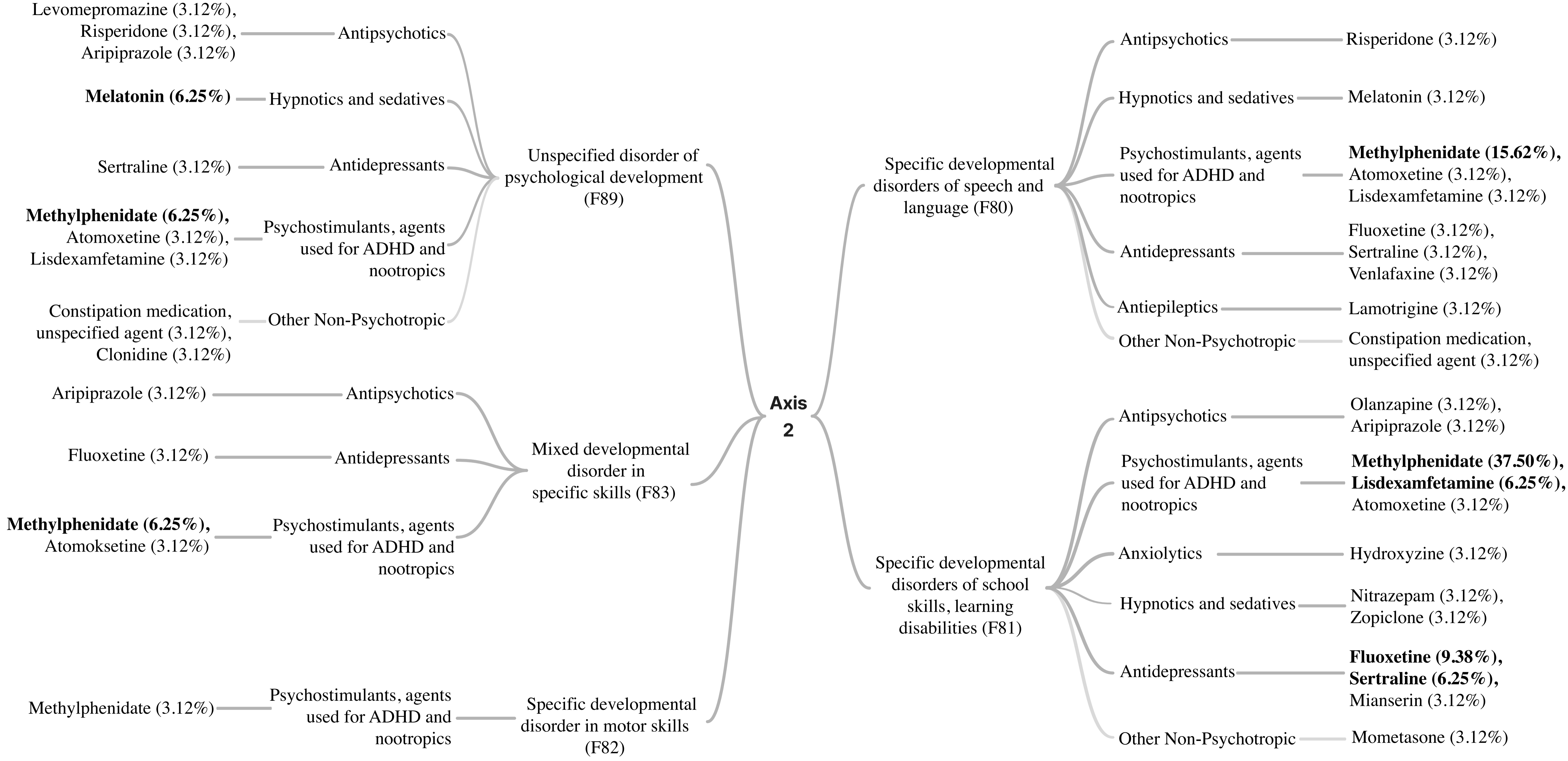

Supplement: Multimedia Appendix 9 [file medinform_v14i1e86066_app9.pdf]
