# Supplementary material for: Mining and Mapping 25 Years of Medication Use in Child and Adolescent Mental Health Services: Contact-Level Descriptive Analysis of Electronic Health Records
Source: JMIR Med Inform. 2026 Jun 16;14:e86066. doi: 10.2196/86066 (PMC13320007; doi:10.2196/86066)

RKBU Data Analytics Tool

Axis 1 Summary | Axis 1 data | Axis 2 Summary | Axis 2 data | Axis 3 Summary | Axis 3 data

Summary: Patient Profile Distributions / Pasientprofildistribusjoner

Filter Options / Filteralternativer

Select demographics to display / Velg demografi:

Age Distribution × | Gender Distribut… × | Mother Relation… × | Father Relations… × | Mother Ethnicity × | Father Ethnicity × | Home Language ×

Age Distribution (Unique Patients)

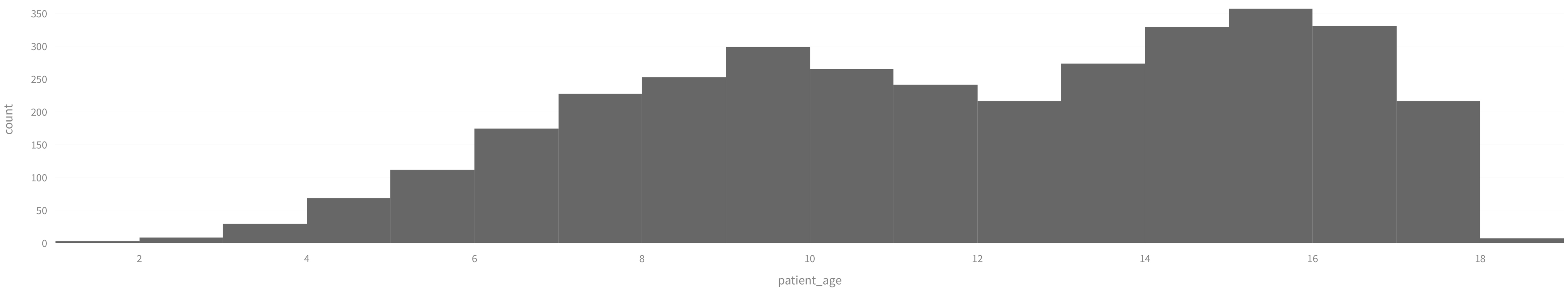

Gender Distribution

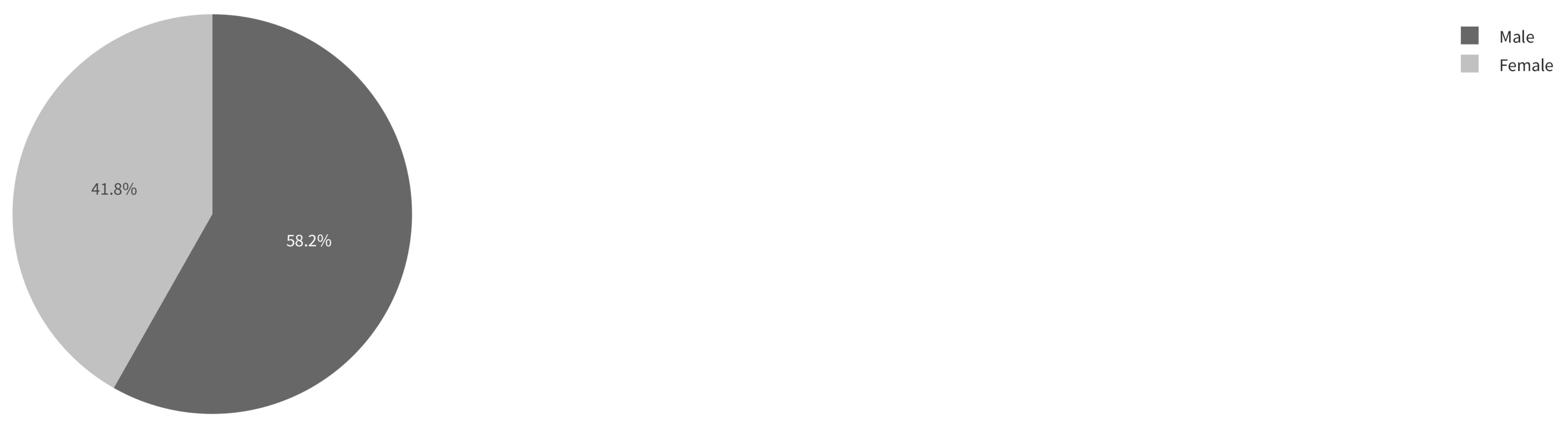

Mother Relationship Distribution

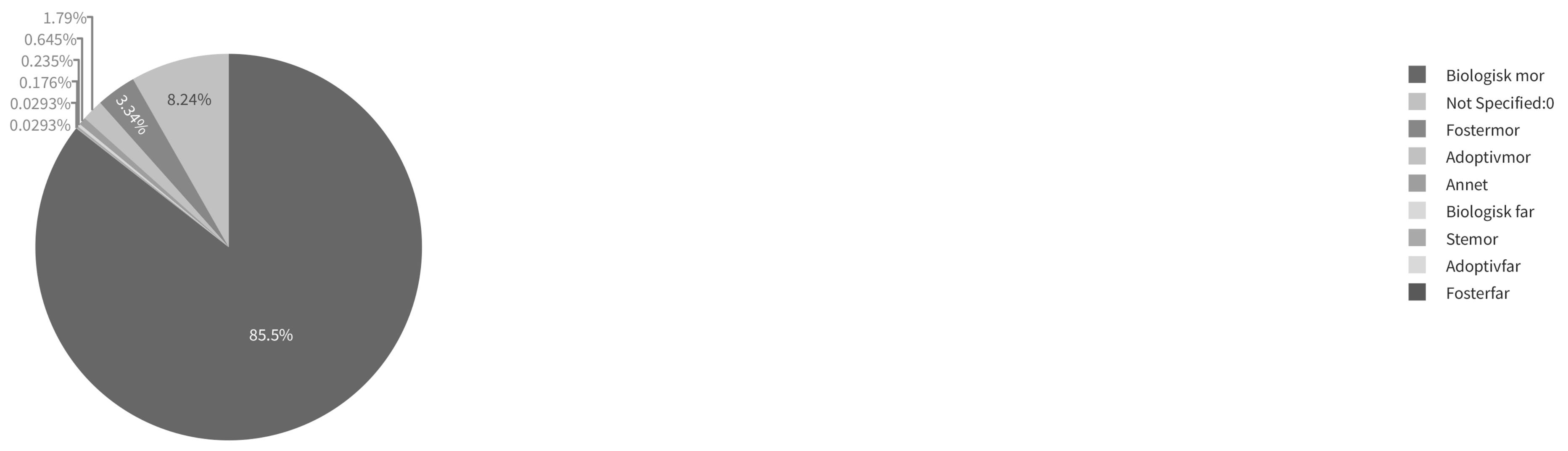

Father Relationship Distribution

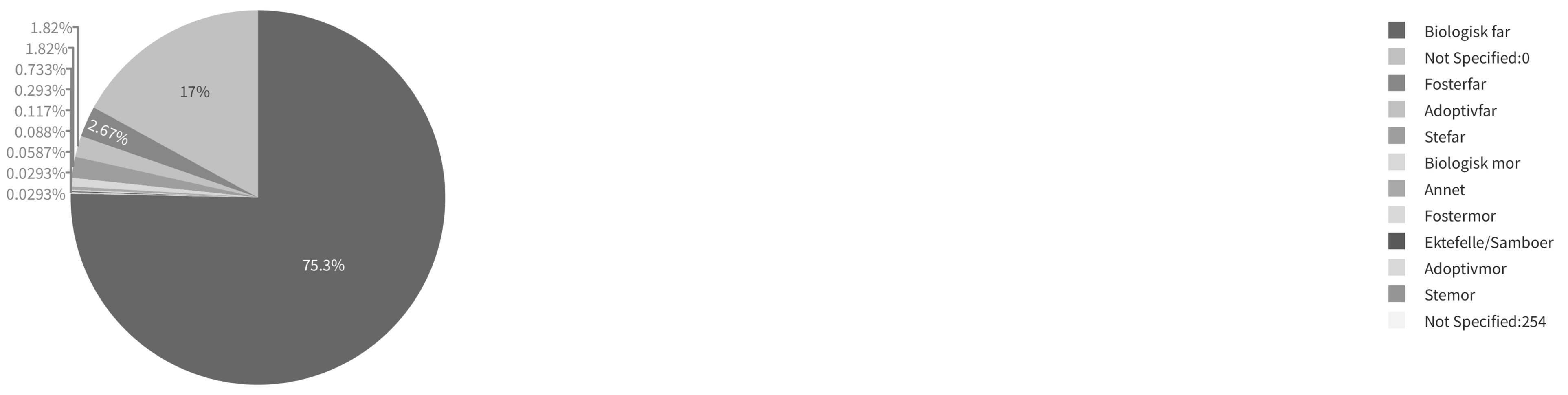

Mother Ethnicity Distribution

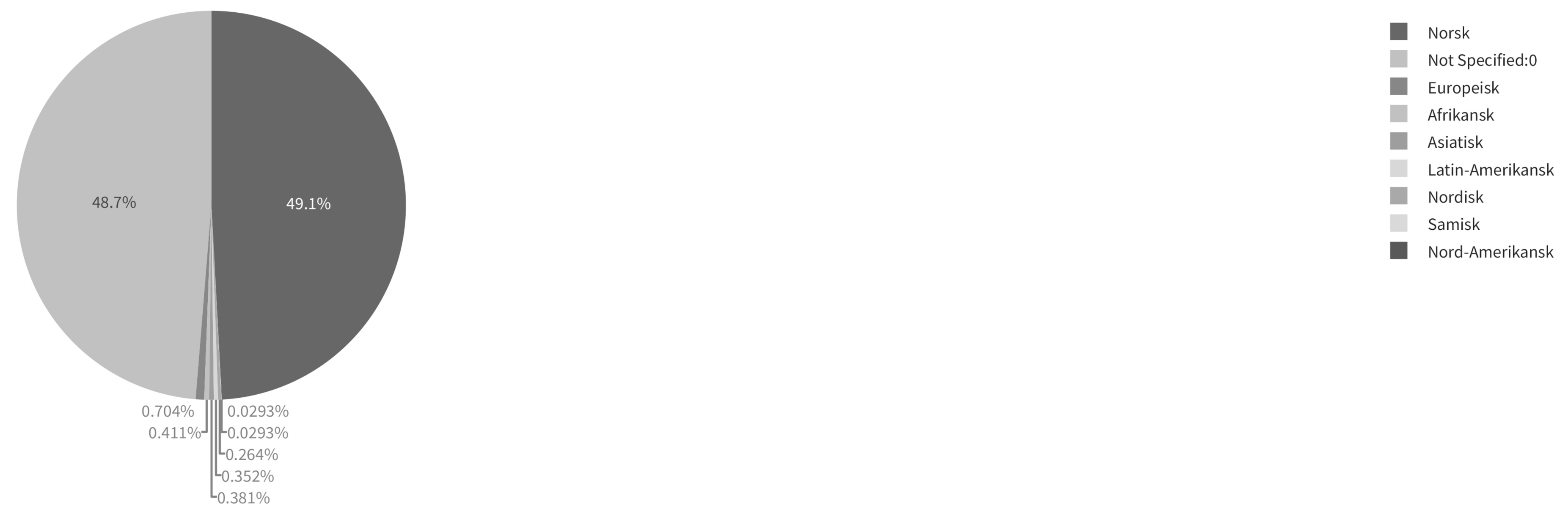

Father Ethnicity Distribution

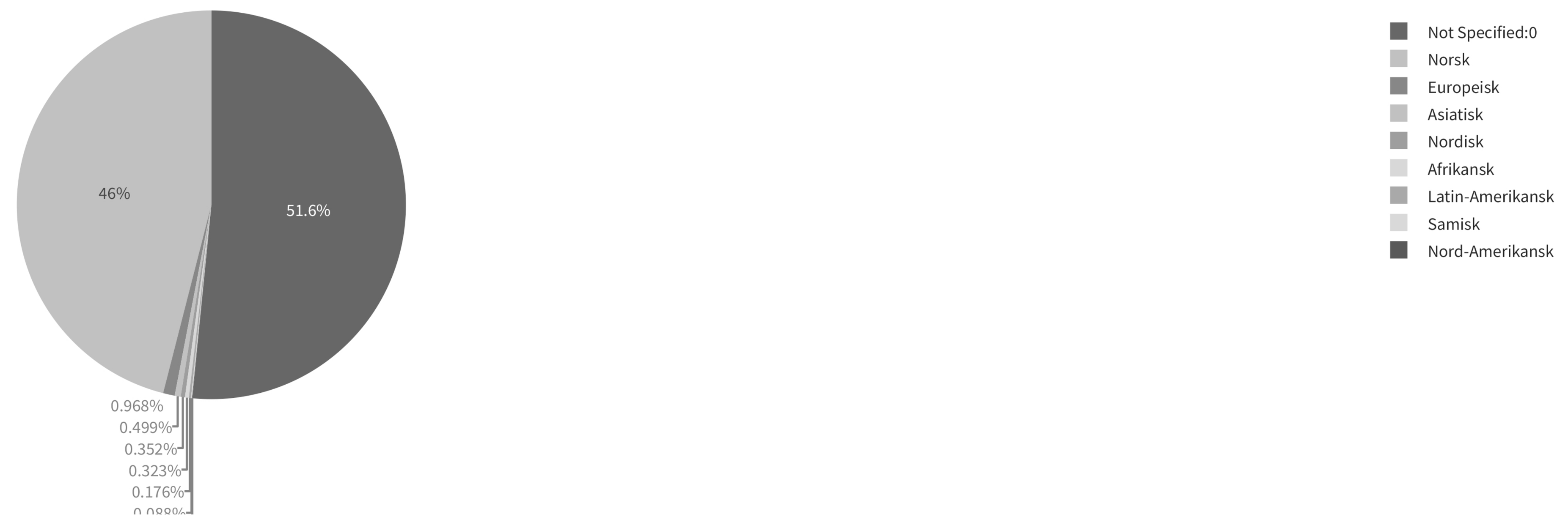

Home Language Distribution

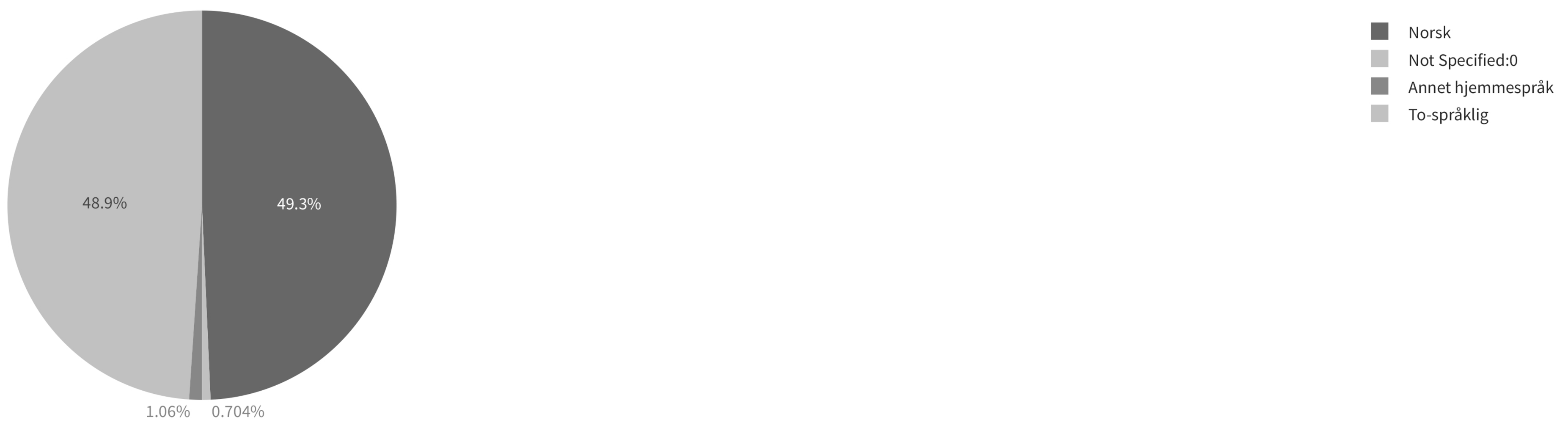

RKBU Data Analytics Tool

Axis 1 Summary | Axis 1 data | Axis 2 Summary | Axis 2 data | Axis 3 Summary | Axis 3 data

Axis 1 Analysis

Filter Options

- [x] Apply ICD Filter

Select ICD Code

- [ ] Apply ATC Filter
- [ ] Apply Gender Filter
- [ ] Apply Age Filter

Patient Overview

Axis 1 Patients: 3410 | Episodes: 4682 | Contacts: 7179

Distribution of Contacts per Patient

Contacts per Patient

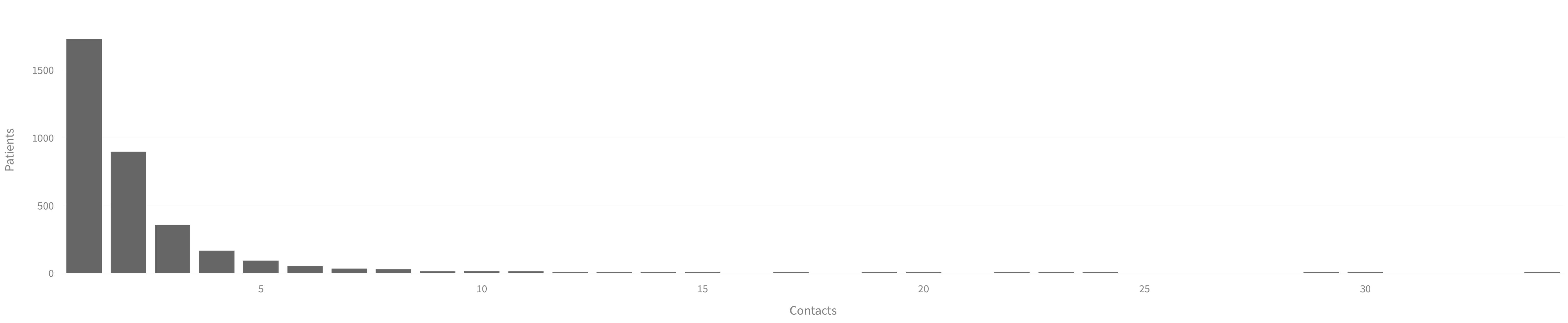

Diagnosis Table

	diag_diagnose	diag_akse	patient_count	↓ contact_count
125	F900	1	1,989	2,819
173	Z032	1	573	815
126	F901	1	216	393
48	F321	1	214	371
123	F845	1	118	208
150	F952	1	133	194
82	F431	1	102	170
100	F500	1	55	156
50	F322	1	67	117
124	F849	1	64	107

ATC Table

	atckode	patient_count	↓ contact_count	ATC_Name
100	N06BA04	2,444	4,604	Metylfenidat
93	N06AB06	388	1,275	Sertralin
101	N06BA09	433	937	Atomoksetin
90	N06AB03	301	922	Fluoxetin
10	A06BA04	510	865	None
111	R06AD01	270	827	Alimemazin
87	N05CH01	367	758	Melatonin
78	N05AH04	160	708	Quetiapin
79	N05AX08	259	677	Risperidon
102	N06BA12	309	555	Lisdeksamfetamin

Primary Diagnoses per Contacts

	pasient_nr	opphold_id	diagnoses	names
0	16	1,042	F900	F900
1	19	14,277	F900	F900
2	23	32,058	F900	F900
3	24	29,291	F900	F900
4	28	18,788	Z032	Z032
5	45	5,940	F900	F900
6	45	34,251	F900	F900
7	51	7,674	F900	F900
8	64	11,600	F459	F459
9	66	22,512	F845	F845

Patient Trajectory by Contact

Page

1

Patient Trajectory by Contact

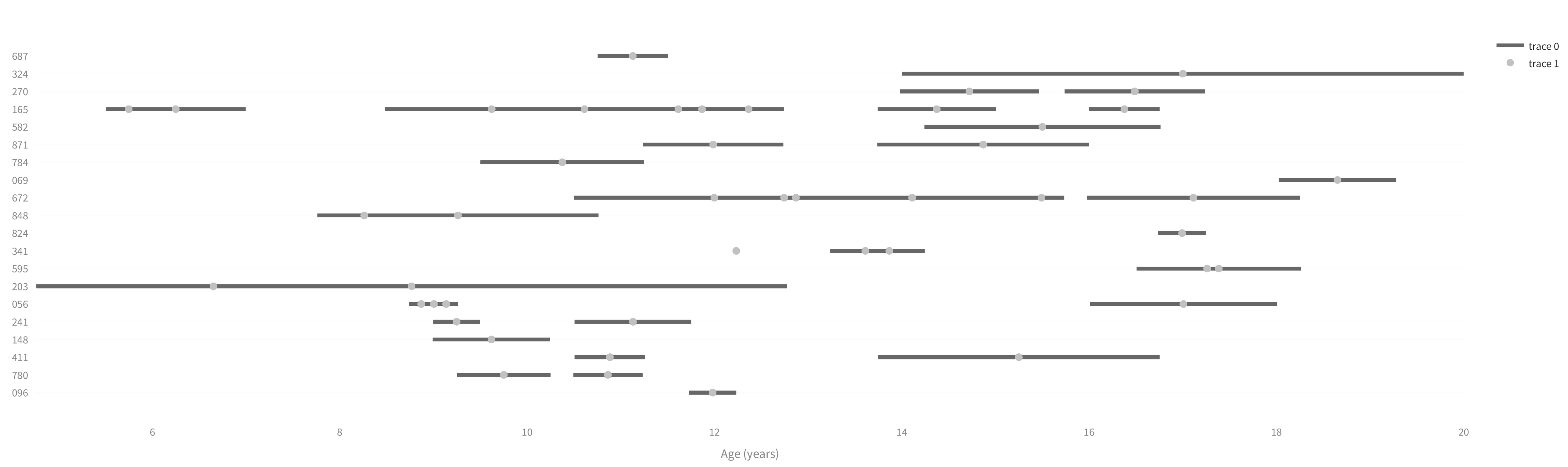

RKBU Data Analytics Tool

Axis 2 Analysis

Filter Options

Patient Overview

Axis 2 Patients: 546 | Episodes: 644 | Contacts: 821

Distribution of Contacts per Patient

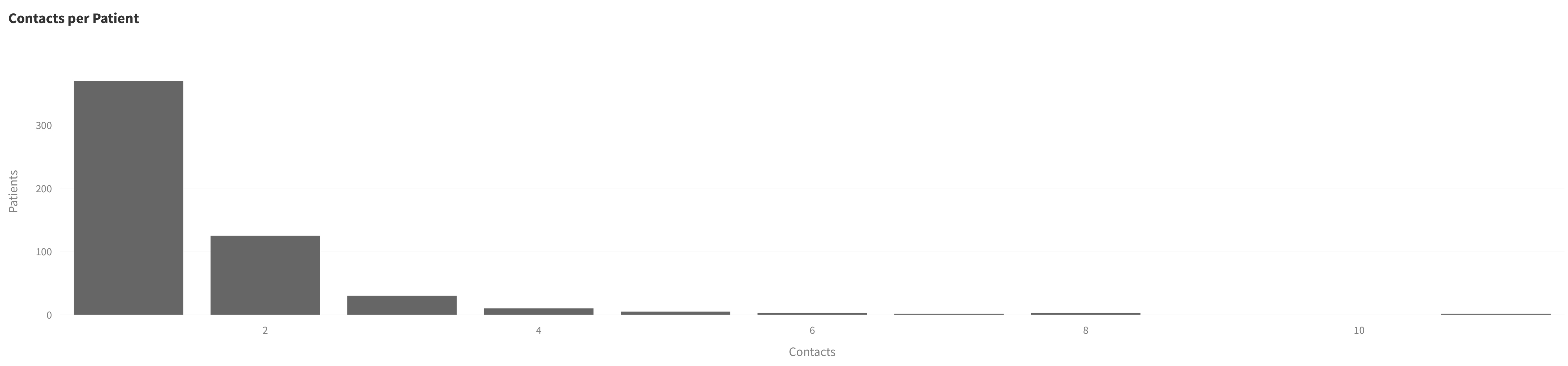

Diagnosis Table

	diag_diagnose	diag_akse	patient_count	contact_count
0	F80	2	10	13
1	F800	2	16	23
2	F801	2	37	53
3	F802	2	22	42
4	F808	2	11	14
5	F809	2	19	29
6	F81	2	1	1
7	F810	2	153	198
8	F811	2	16	24
9	F812	2	16	24

ATC Table

	atckode	patient_count	contact_count	ATC_Name
0	A03FA01	1	2	Metoklopramid
1	A06AD65	1	2	Makrogol, kombinasjoner
2	A06BA04	89	111	None
3	A10BA02	1	4	Metformin
4	A11EA	5	8	Vitamin B-kompleks, usan
5	A12AX	1	2	Kalsium, kombinasjoner n
6	A12BA02	1	2	Kaliumsitrat
7	B03AA07	1	1	Ferrosulfat
8	C02AC02	1	1	None
9	D01AC20	1	7	Kombinasjoner

Primary Diagnoses per Contacts

	pasient_nr	opphold_id	diagnoses	names
0	122	13,079	F810	F810
1	145	23,882	F801	F801
2	154	37,437	F813	F813
3	156	1,912	F819	F819
4	178	14,652	F83	F83
5	212	12,389	F83	F83
6	212	31,533	F813	F813
7	240	35,565	F819	F819
8	276	34,449	F801	F801
9	311	27,966	F813	F813

Patient Trajectory by Contact

Page

1

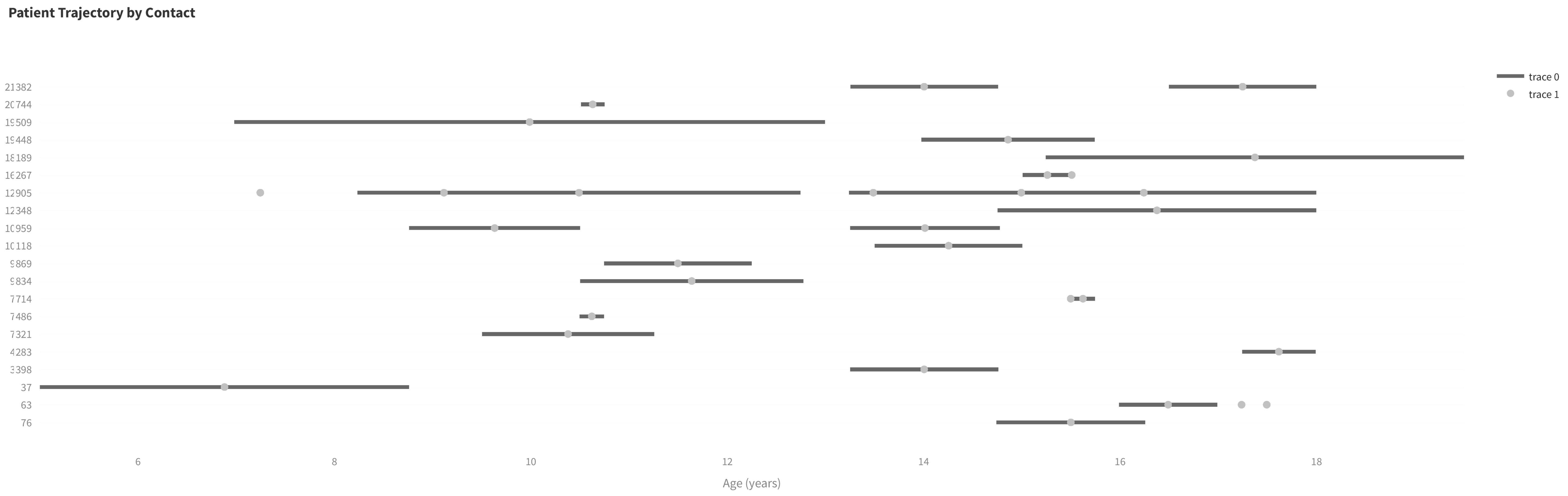

RKBU Data Analytics Tool

Axis 1 Summary | Axis 1 data | Axis 2 Summary | Axis 2 data | Axis 3 Summary | Axis 3 data

Axis 3 Analysis

Filter Options

Patient Overview

Axis 3 Patients: 37 | Episodes: 46 | Contacts: 65

Distribution of Contacts per Patient

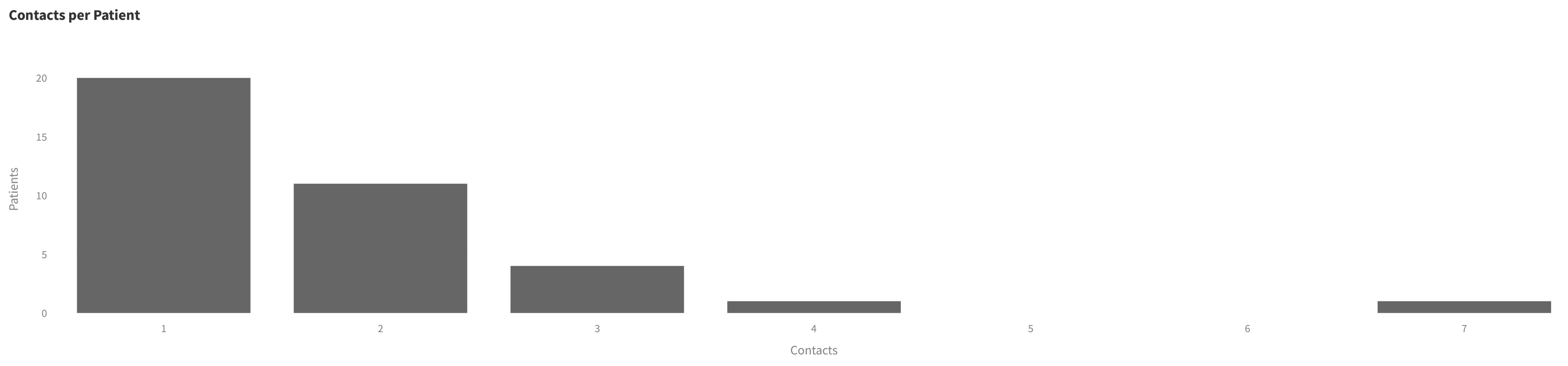

Diagnosis Table

	diag_diagnose	diag_akse	patient_count	contact_count
0	F700	3	18	24
1	F701	3	10	19
2	F708	3	6	10
3	F709	3	1	2
4	F710	3	2	3
5	F711	3	2	3
6	F719	3	2	2
7	F791	3	1	2

ATC Table

	atckode	patient_count	contact_count	ATC_Name
0	A06AD11	1	1	Laktulose
1	A06BA04	2	5	None
2	A10BA02	1	3	Metformin
3	C02AC02	1	1	None
4	G03AC06	1	1	Medroksyprogesteron
5	N02CX02	1	4	Klonidin
6	N03AG01	1	3	Valproinsyre
7	N03AX09	1	1	Lamotrigin
8	N05AA02	7	19	Levomepromazin
9	N05AF03	1	1	Klorprotiksen

Primary Diagnoses per Contacts

	pasient_nr	opphold_id	diagnoses	names
0	358	8,178	F710	F710
1	1,750	1,514	F700	F700
2	2,198	27,616	F700	F700
3	3,237	11,359	F719	F719
4	3,237	37,965	F711	F711
5	3,864	21,508	F701	F701
6	3,878	2,196	F701	F701
7	3,878	38,240	F701	F701
8	4,407	14,290	F700	F700
9	4,407	20,662	F700	F700

Patient Trajectory by Contact

Page

1

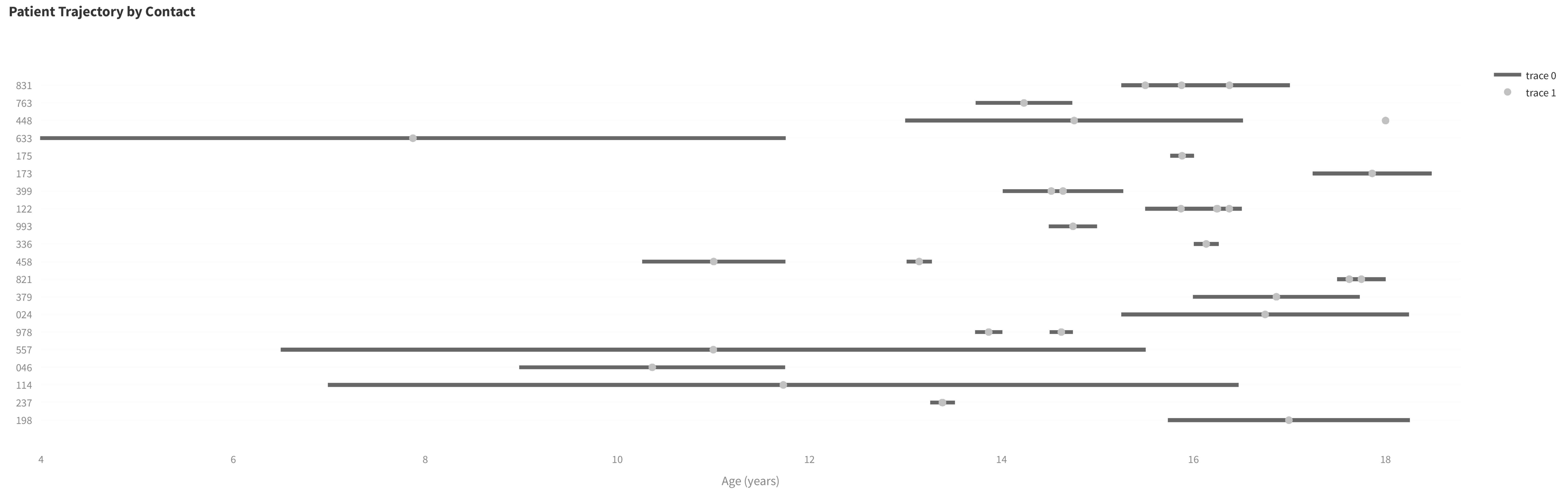

RKBU Data Analytics Tool

Axis 1 Summary | Axis 1 data | Axis 2 Summary | Axis 2 data | Axis 3 Summary | Axis 3 data

Axis 1 Analysis

Filter Options

- [x] Apply ICD Filter

Select ICD Code

F900

- [ ] Apply ATC Filter
- [ ] Apply Gender Filter
- [ ] Apply Age Filter

Patient Overview

Axis 1 Patients: 1989 | Episodes: 2358 | Contacts: 2819

Distribution of Contacts per Patient

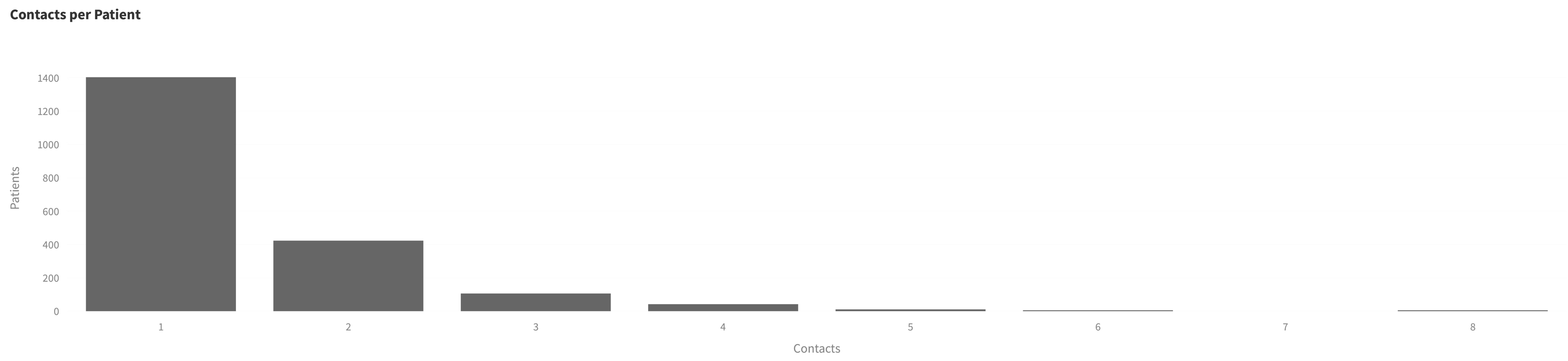

Diagnosis Table

	diag_diagnose	diag_akse	patient_count	↓ contact_coun
0	F900	1	1,989	2,819

ATC Table

	atckode	patient_count	↓ contact_coun	ATC_Name
55	N06BA04	1,892	2,673	Metylfenidat
4	A06BA04	403	546	None
56	N06BA09	304	501	Atomoksetin
57	N06BA12	234	347	None
44	N05CH01	169	238	Melatonin
38	N05AX08	101	193	Risperidon
62	R06AD01	51	82	Alimemazin
46	N06AB03	42	74	Fluoxetin
48	N06AB06	42	61	Sertralin
39	N05AX12	26	47	Aripiprazol

Primary Diagnoses per Contacts

	pasient_nr	opphold_id	diagnoses	names
0	16	1,042	F900	F900
1	19	14,277	F900	F900
2	23	32,058	F900	F900
3	24	29,291	F900	F900
4	45	5,940	F900	F900
5	45	34,251	F900	F900
6	51	7,674	F900	F900
7	82	36,453	F900	F900
8	89	20,582	F900	F900
9	89	33,107	F900	F900

Patient Trajectory by Contact

Page

1

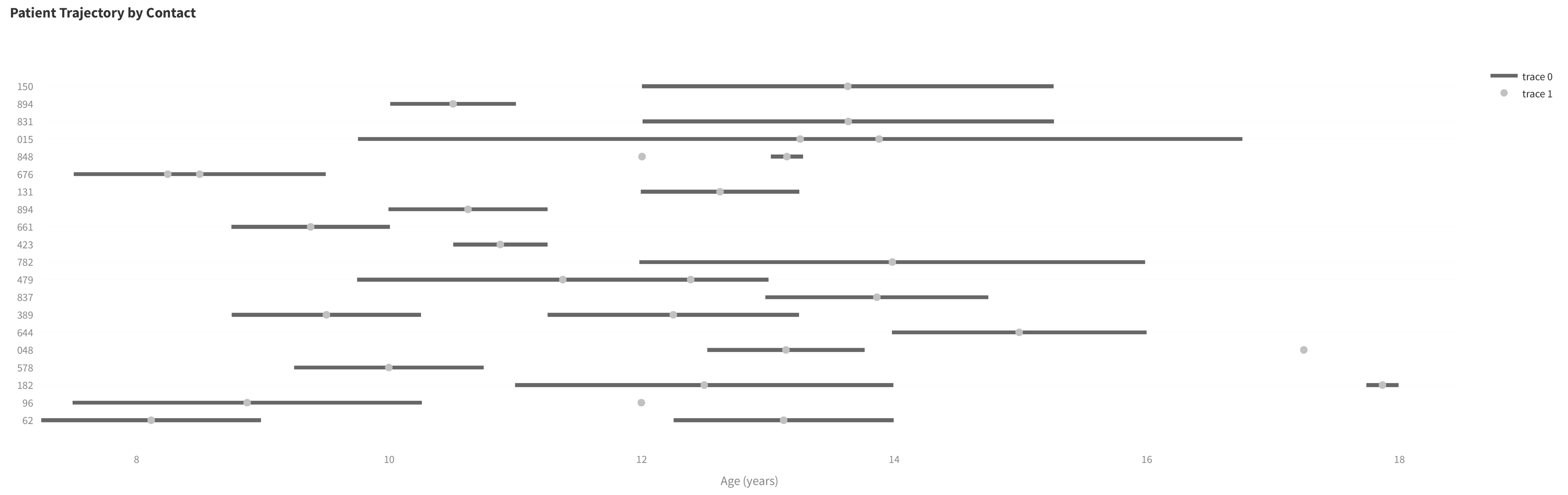

Supplement: Multimedia Appendix 3 [file medinform_v14i1e86066_app3.pdf]
