# Supplementary material for: Mining and Mapping 25 Years of Medication Use in Child and Adolescent Mental Health Services: Contact-Level Descriptive Analysis of Electronic Health Records
Source: JMIR Med Inform. 2026 Jun 16;14:e86066. doi: 10.2196/86066 (PMC13320007; doi:10.2196/86066)

(a) Complete axis I diagnosis and medications mapping for all contacts

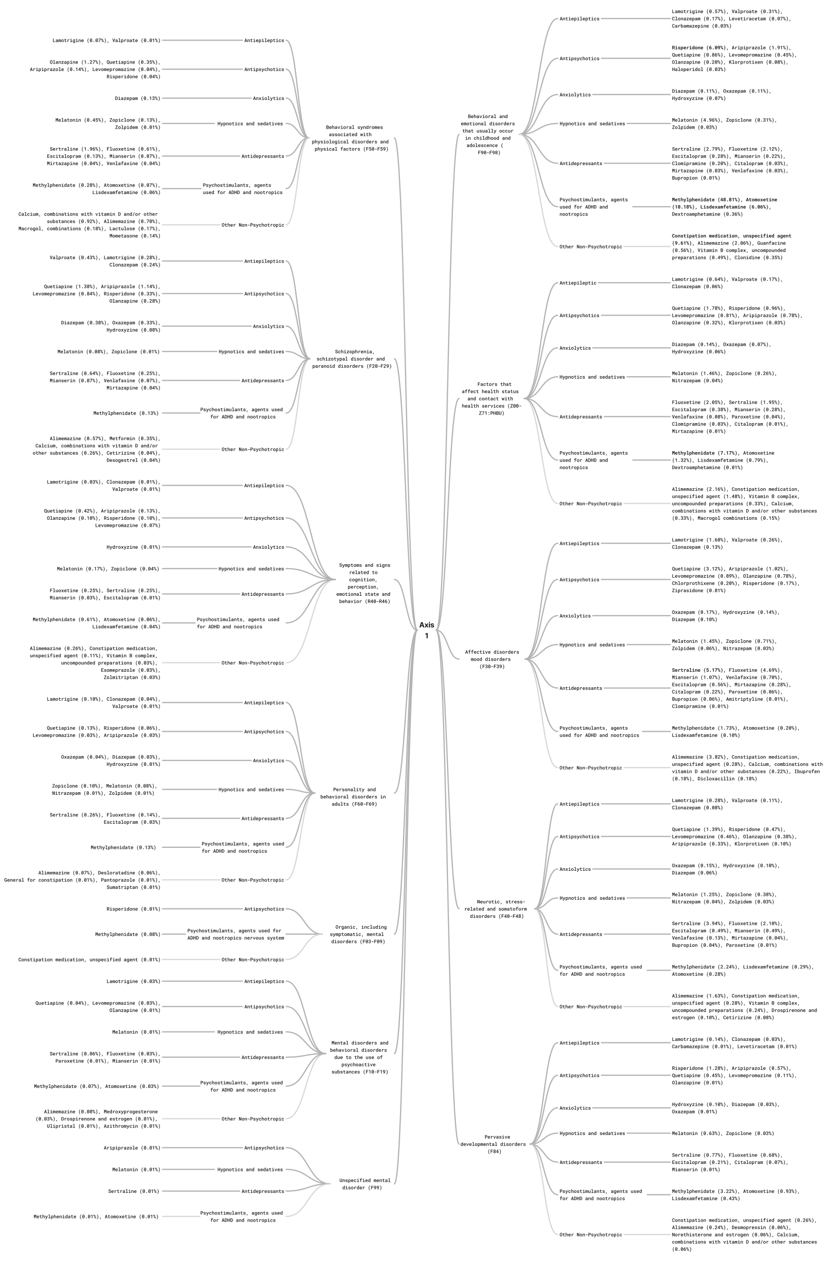

(b) Complete axis I diagnosis and medications mapping for non-comorbid contacts

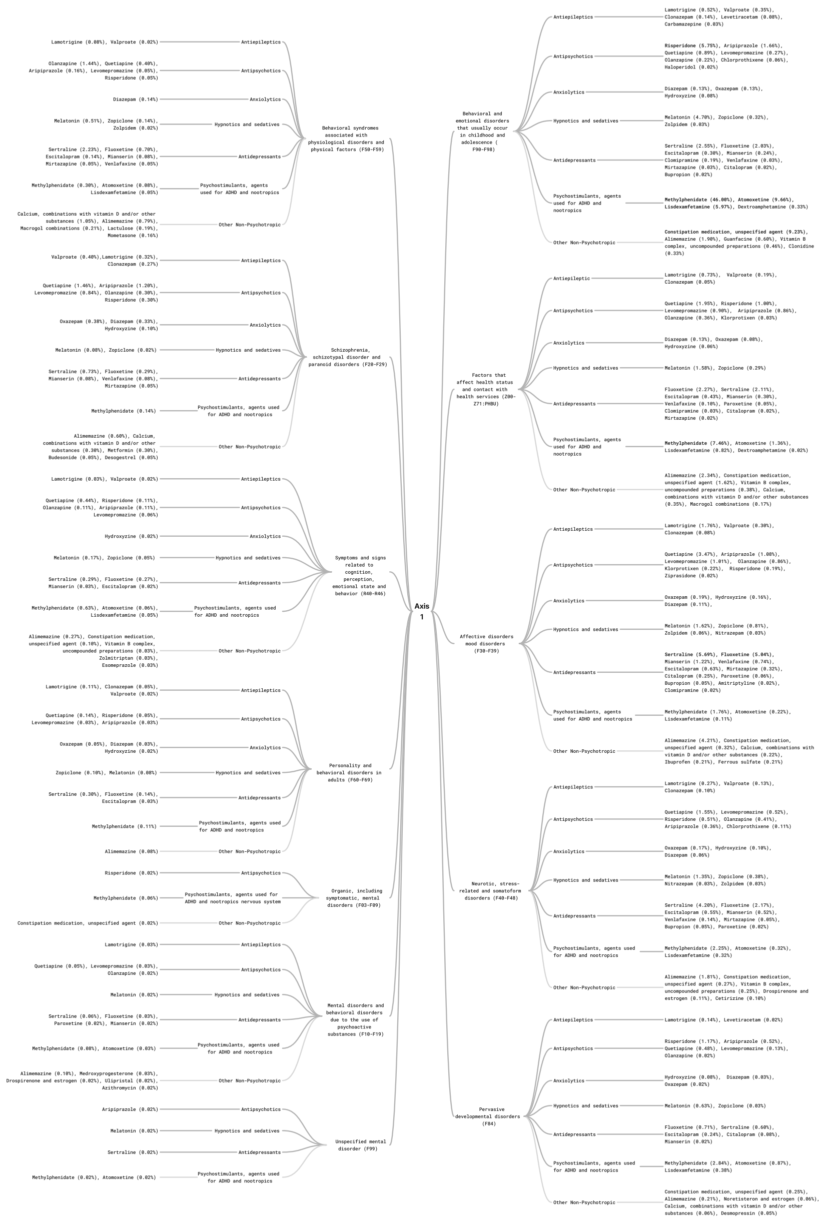

Supplement: Multimedia Appendix 8 [file medinform_v14i1e86066_app8.pdf]
