# Supplementary material for: Mining and Mapping 25 Years of Medication Use in Child and Adolescent Mental Health Services: Contact-Level Descriptive Analysis of Electronic Health Records
Source: JMIR Med Inform. 2026 Jun 16;14:e86066. doi: 10.2196/86066 (PMC13320007; doi:10.2196/86066)
Supplement: Multimedia Appendix 10 [file medinform_v14i1e86066_app10.pdf]

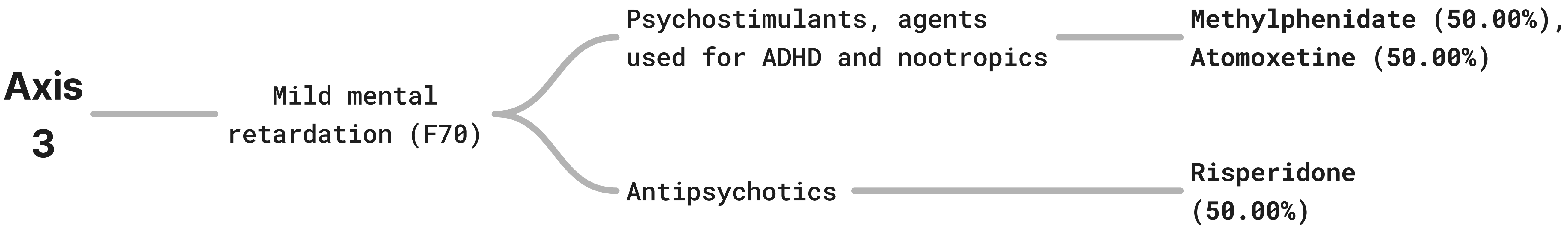
Axis 3
Mild mental retardation (F70)
Psychostimulants, agents used for ADHD and nootropics
Methylphenidate (50.00%), Atomoxetine (50.00%)
Antipsychotics
Risperidone (50.00%)
