# Supplementary material for: Mining and Mapping 25 Years of Medication Use in Child and Adolescent Mental Health Services: Contact-Level Descriptive Analysis of Electronic Health Records
Source: JMIR Med Inform. 2026 Jun 16;14:e86066. doi: 10.2196/86066 (PMC13320007; doi:10.2196/86066)
Supplement: Multimedia Appendix 11 [file medinform_v14i1e86066_app11.pdf]

Demographic characteristic	Value
**Cohort**	Total patients (N): 546; Total episodes of care: 644; Total contacts (C2): 821
**Axis 2 patient-level contact distribution summary (N)**	Median contacts per patient: 1; Range (Min–max contacts per patient): [1–11];<br>Contacts per patient IQR: [1–2]; Number of patients in top 5%: 28;<br>Contacts contributed by top 5%: 132; Share of all contacts from top 5%: 16.08%
**Comorbid and non-comorbid contacts**	Total comorbid contacts: 789; Total non-comorbid contacts: 32
**Gender (N)**	Male: 365 (66.85%); Female: 181 (33.15%)
**Episodes of care start**	Oldest: 1999-10-05; Newest: 2018-01-04
**Episodes of care end**	Oldest: 2002-04-05; Newest: 2019-04-01
**Age at first episode (N)**	Mean: 11; SD: 3; Median: 11; IQR: [9 –14]; Range: [2–18]
**Home language (N)**	Not specified: 212 (38.83%); Norwegian: 326 (59.71%)<br>Other: 2 (0.37%); Bilingual: 6 (1.10%)
**Mothers relation (N)**	Not specified: 24 (4.40%); Biological mother: 480 (87.91%)<br>Biological father: 1 (0.18%); Adoptive mother: 13 (2.38%)<br>Foster mother: 23 (4.21%); Stepmother: 1 (0.18%)<br>Adoptive father: 1 (0.18%); Other: 3 (0.55%)
**Fathers relation (N)**	Not specified: 63 (11.54%); Biological father: 426 (78.02%)<br>Biological mother: 6 (1.10%); Adoptive father: 14 (2.56%)<br>Stepfather: 13 (2.38%); Foster father: 21 (3.85%)<br>Spouse/Partner: 2 (0.37%); Other: 1 (0.18%)
**Mothers ethnicity (N)**	Not specified: 212 (38.83%); Norwegian: 322 (58.97%); Sami: 1 (0.18%);<br>Nordic: 1 (0.18%); European: 3 (0.55%); Asian: 2 (0.37%)<br>African: 3 (0.55%); Latin-American: 2 (0.37%)
**Fathers ethnicity (N)**	Not specified: 231 (42.31%); Norwegian: 301 (55.13%); Sami: 2 (0.37%);<br>Nordic: 2 (0.37%); European: 6 (1.10%); Asian: 3 (0.55%); African: 1 (0.18%)
**Total diagnoses and medications**	Diagnoses: 17; Medications: 57
**Most frequent 5 diagnoses (C2)**	F810: Specific reading disorder (24.1%)<br>F813: Mixed developmental disorder in school ability (21.8%)<br>F83: Mixed developmental disorder in specific skills (12.4%)<br>F82: Specific developmental disorder in motor skills (7.2%)<br>F801: Expressive language disorder (6.5%)
**Most frequent 5 medications (C2)**	N06BA04: Methylphenidate (84.7%); N06BA09: Atomoxetine (15.2%);<br>A06BA04: Constipation medications, unspecified agent (13.5%);<br>N06AB06: Sertraline (10.1%); N05CH01: Melatonin (8.9%)
