# Supplementary material for: Mining and Mapping 25 Years of Medication Use in Child and Adolescent Mental Health Services: Contact-Level Descriptive Analysis of Electronic Health Records
Source: JMIR Med Inform. 2026 Jun 16;14:e86066. doi: 10.2196/86066 (PMC13320007; doi:10.2196/86066)
Supplement: Multimedia Appendix 12 [file medinform_v14i1e86066_app12.pdf]

Demographic Characteristic	Value
**Cohort**	Total patients (N): 37; Total episodes of care: 46<br>Total contacts (C3): 65
**Axis 3 patient-level contact distribution summary (N)**	Median contacts per patient: 1; Range (Min–max contacts per patient): [1–7];<br>Contacts per patient IQR: [1–2]; Number of patients in top 5%: 2;<br>Contacts contributed by top 5%: 11; Share of all contacts from top 5%: 16.92%
**Comorbid and non-comorbid contacts**	Total comorbid contacts: 63; Total non-comorbid contacts: 2
**Gender (N)**	Male: 26 (70.27%); Female: 11 (29.73%)
**Episodes of Care Start**	Oldest: 2000-01-07; Newest: 2017-01-04
**Episodes of Care End**	Oldest: 2009-03-30; Newest: 2017-10-06
**Age at first episode (N)**	Mean: 13; SD: 4; Median: 15; IQR: [13 –16]; Range: [4–18]
**Home Language (N)**	Not Specified: 11 (29.73%); Norwegian: 26 (70.27%)
**Mothers Relation (N)**	Not Specified: 2 (5.41%); Biological mother: 27 (72.97%)<br>Adoptive mother: 1 (2.70%); Stepmother: 1 (2.70%)<br>Foster mother: 6 (16.22%)
**Fathers Relation (N)**	Not Specified: 6 (16.22%); Biological father: 25 (67.57%)<br>Adoptive father: 1 (2.70%); Foster father: 5 (13.51%)
**Mothers Ethnicity (N)**	Not Specified: 9 (24.32%); Norwegian: 27 (72.97%)<br>African: 1 (2.70%)
**Fathers Ethnicity (N)**	Not Specified: 13 (35.14%); Norwegian: 24 (64.86%)
**Total Diagnoses and Medications**	Diagnoses codes: 8; Medications codes: 24
**Most Frequent 5 Diagnoses (C3)**	F700: Mild mental retardation with no or minimal behavioral problem (36.9%)<br>F701: Mild mental retardation with significant behavioral problem (29.2%)<br>F708: Mild mental retardation with other indication of behavioral problem (15.4%)<br>F710: Moderate mental retardation with no or minimal behavioral problem (4.6%)<br>F711: Moderate mental retardation with significant behavioral problem (4.6%)
**Most Frequent 5 Medications (C3)**	N05AX08: Risperidone (55.4%); N06BA04: Methylphenidate (53.8%);<br>N05AA02: Levomepromazine (29.2%); N06BA09: Atomoxetine (27.7%);<br>N05AX12: Aripiprazole (23.1%)
