# Supplementary material for: Mining and Mapping 25 Years of Medication Use in Child and Adolescent Mental Health Services: Contact-Level Descriptive Analysis of Electronic Health Records
Source: JMIR Med Inform. 2026 Jun 16;14:e86066. doi: 10.2196/86066 (PMC13320007; doi:10.2196/86066)
Supplement: Multimedia Appendix 14 [file medinform_v14i1e86066_app14.pdf]

Medication Category	Medications and approval date in Norway
Antipsychotics	Risperidone (1994, September 22), Aripiprazole (2004, June 24),<br>Quetiapine (2000, December 21), Levomepromazine (1960, January 15),<br>Olanzapine (1997, February 24)
Anxiolytics	Diazepam (1963, September 17), Oxazepam (1966, November 4),<br>Hydroxyzine (1957, October 25)
Hypnotics & Sedatives	Melatonin (2007, June 29), Zopiclone (1994, July 6),<br>Zolpidem (1996, April 29), Nitrazepam (1965, July 23)
Antidepressants	Sertraline (1996, May 6), Fluoxetine (1995, December 12),<br>Escitalopram (2002, June 27), Mianserin (1982, January 15),<br>Venlafaxine (1996, July 4)
Psychostimulants & ADHD Agents	Methylphenidate (1956, November 30), Atomoxetine (2004, November 25),<br>Lisdexamfetamine (2013, February 21), Dextroamphetamine (2014, October 9)
Antiepileptics	Lamotrigine (1994, January 13), Valproate (1980, April 12),<br>Levetiracetam (2000, September 29), Clonazepam (1974, November 20),<br>Carbamazepine (1965, February 18)
Other Non-Psychotropic	Constipation medications, unspecified agent (Not available), Alimemazine (1960, August 26),<br>Calcium & vitamin D combos (1989, May 25), Vitamin B complex (1938, June 17),<br>Desmopressin (1979, February 9), Drospirenon & estrogen (2000, November 27)
