# Supplementary material for: Mining and Mapping 25 Years of Medication Use in Child and Adolescent Mental Health Services: Contact-Level Descriptive Analysis of Electronic Health Records
Source: JMIR Med Inform. 2026 Jun 16;14:e86066. doi: 10.2196/86066 (PMC13320007; doi:10.2196/86066)
Supplement: Multimedia Appendix 13 [file medinform_v14i1e86066_app13.pdf]

**In Axis I, 866 comorbid contacts (C)**										
Primary Category	Rank#1	Rank#2	Rank#3	Rank#4	Rank#5	Rank#6	Rank#7	Rank#8	Rank#9	Rank#10
Axis1:F03–F09<br>Organic, symptomatic	Axis2:F82<br>C: 0.23%									
Axis1:F20–F29<br>Schizophrenia & paranoias	Axis3:F70<br>C: 0.69%	Axis2:F83<br>C: 0.46%	Axis2:F81<br>C: 0.23%	Axis1:F710<br>C: 0.12%	Axis3:F71<br>C: 0.12%					
Axis1:F30–F39<br>Affective/mood disorders	Axis2:F81<br>C: 1.85%	Axis1:F30–F39<br>C: 1.15%	Axis2:F80<br>C: 0.69%	Axis1:F40–F48<br>C: 0.23%	Axis1:F84<br>C: 0.12%	Axis2:F83<br>C: 0.12%	Axis3:F70<br>C: 0.12%			
Axis1:F40–F48<br>Neurotic & somatoform	Axis2:F81<br>C: 3.12%	Axis2:F83<br>C: 0.69%	Axis2:F80<br>C: 0.58%	Axis1:F30–F39<br>C: 0.23%	Axis1:Z00–Z71<br>C: 0.23%	Axis1:F90–F98<br>C: 0.12%	Axis2:F82<br>C: 0.12%	Axis2:F89<br>C: 0.12%		
Axis1:F50–F59<br>Behavioral syndromes	Axis1:F90–F98<br>C: 0.12%									
Axis1:F60–F69<br>Personality disorders	Axis1:F60–F69<br>C: 0.23%	Axis2:F80<br>C: 0.12%								
Axis1:F84<br>Pervasive developmental	Axis2:F81<br>C: 3.81%	Axis2:F83<br>C: 1.62%	Axis2:F80<br>C: 1.27%	Axis2:F82<br>C: 0.81%	Axis3:F70<br>C: 0.58%	Axis1:F84<br>C: 0.46%	Axis3:F71<br>C: 0.23%	Axis1:F30–F39<br>C: 0.12%	Axis1:R40–R46<br>C: 0.12%	
Axis1:F90–F98<br>Childhood emotional disorders	**Axis2:F81<br>C: 41.11%**	**Axis2:F80<br>C: 15.24%**	**Axis2:F83<br>C: 8.20%**	Axis2:F82<br>C: 4.97%	Axis3:F70<br>C: 4.27%	Axis1:F90–F98<br>C: 3.46%	Axis1:Z00–Z71<br>C: 0.81%	Axis2:F89<br>C: 0.35%	Axis2:F88<br>C: 0.23%,<br>Axis3:F71<br>C: 0.23%,<br>Axis3:F79<br>C: 0.23%	Axis1:F50–F59<br>C: 0.12%,<br>Axis1:F40–F48<br>C: 0.12%
Axis1:R40–R46<br>Cognition & emotional signs	Axis2:F81<br>C: 0.46%	Axis1:F710<br>C: 0.12%	Axis1:F84<br>C: 0.12%							
Axis1:Z00–Z71<br>Health services contacts	Axis2:F81<br>C: 3.35%	Axis2:F80<br>C: 1.27%	Axis1:F90–F98<br>C: 0.81%	Axis2:F82<br>C: 0.58%	Axis2:F83<br>C: 0.35%	Axis3:F70<br>C: 0.35%	Axis1:F40–F48<br>C: 0.23%	Axis1:Z00–Z71<br>C: 0.23%	Axis1:F710<br>C: 0.12%	
**In Axis II, 789 comorbid contacts (C)**										
Axis2:F80<br>Speech & language	**Axis1:F90–F98<br>C: 16.73%**	Axis3:F70<br>C: 1.39%	Axis1:F84<br>C: 1.39%	Axis1:Z00–Z71<br>C: 1.39%	Axis1:F30–F39<br>C: 0.76%	Axis1:F40–F48<br>C: 0.63%	Axis2:F80<br>C: 0.25%	Axis1:F60–F69<br>C: 0.13%,<br>Axis2:F81<br>C: 0.13%		
Axis2:F81<br>School skills & learning	**Axis1:F90–F98<br>C: 45.12%**	Axis1:F84<br>C: 4.18%	Axis1:Z00–Z71<br>C: 3.68%	Axis1:F40–F48<br>C: 3.42%	Axis1:F30–F39<br>C: 2.03%	Axis1:R40–R46<br>C: 0.51%	Axis1:F20–F29<br>C: 0.25%	Axis2:F80<br>C: 0.13%,<br>Axis2:F83<br>C: 0.13%,<br>Axis3:F70<br>C: 0.13%		
Axis2:F82<br>Motor skills	**Axis1:F90–F98<br>C: 5.45%**	Axis1:F84<br>C: 0.89%	Axis1:Z00–Z71<br>C: 0.63%	Axis3:F70<br>C: 0.38%	Axis1:F03–F09<br>C: 0.25%	Axis1:F40–F48<br>C: 0.13%				
Axis2:F83<br>Mixed specific skills	**Axis1:F90–F98<br>C: 9.00%**	Axis1:F84<br>C: 1.77%	Axis1:F40–F48<br>C: 0.76%	Axis1:F20–F29<br>C: 0.51%	Axis1:Z00–Z71<br>C: 0.38%	Axis1:F30–F39<br>C: 0.13%,<br>Axis2:F81<br>C: 0.13%,<br>Axis3:F70<br>C: 0.13%				
Axis2:F88<br>Other psychological dev	Axis1:F90–F98<br>C: 0.25%									
Axis2:F89<br>Unspecified psych dev	Axis1:F90–F98<br>C: 0.38%	Axis1:F40–F48<br>C: 0.13%								
**In Axis III, 63 comorbid contacts (C)**										
Axis3:F70<br>Mild mental retardation	**Axis1:F90–F98<br>C: 58.73%**	**Axis2:F80<br>C: 17.46%**	**Axis1:F20–F29<br>C: 9.52%**	**Axis1:F84<br>C: 7.94%**	Axis1:Z00–Z71<br>C: 4.76%	Axis2:F82<br>C: 4.76%	Axis1:F30–F39<br>C: 1.59%,<br>Axis2:F81<br>C: 1.59%,<br>Axis2:F83<br>C: 1.59%			
Axis3:F71<br>Moderate mental retardation	Axis1:F84<br>C: 3.17%	Axis1:F90–F98<br>C: 3.17%	Axis1:F20–F29<br>C: 1.59%							
Axis3:F79<br>Unspecified mental retardation	Axis1:F90–F98<br>C: 3.17%									
